# The Space Physics Environment Data Analysis System (SPEDAS)

**DOI:** 10.1007/s11214-018-0576-4

**Published:** 2019-01-22

**Authors:** V. Angelopoulos, P. Cruce, A. Drozdov, E. W. Grimes, N. Hatzigeorgiu, D. A. King, D. Larson, J. W. Lewis, J. M. McTiernan, D. A. Roberts, C. L. Russell, T. Hori, Y. Kasahara, A. Kumamoto, A. Matsuoka, Y. Miyashita, Y. Miyoshi, I. Shinohara, M. Teramoto, J. B. Faden, A. J. Halford, M. McCarthy, R. M. Millan, J. G. Sample, D. M. Smith, L. A. Woodger, A. Masson, A. A. Narock, K. Asamura, T. F. Chang, C.-Y. Chiang, Y. Kazama, K. Keika, S. Matsuda, T. Segawa, K. Seki, M. Shoji, S. W. Y. Tam, N. Umemura, B.-J. Wang, S.-Y. Wang, R. Redmon, J. V. Rodriguez, H. J. Singer, J. Vandegriff, S. Abe, M. Nose, A. Shinbori, Y.-M. Tanaka, S. UeNo, L. Andersson, P. Dunn, C. Fowler, J. S. Halekas, T. Hara, Y. Harada, C. O. Lee, R. Lillis, D. L. Mitchell, M. R. Argall, K. Bromund, J. L. Burch, I. J. Cohen, M. Galloy, B. Giles, A. N. Jaynes, O. Le Contel, M. Oka, T. D. Phan, B. M. Walsh, J. Westlake, F. D. Wilder, S. D. Bale, R. Livi, M. Pulupa, P. Whittlesey, A. DeWolfe, B. Harter, E. Lucas, U. Auster, J. W. Bonnell, C. M. Cully, E. Donovan, R. E. Ergun, H. U. Frey, B. Jackel, A. Keiling, H. Korth, J. P. McFadden, Y. Nishimura, F. Plaschke, P. Robert, D. L. Turner, J. M. Weygand, R. M. Candey, R. C. Johnson, T. Kovalick, M. H. Liu, R. E. McGuire, A. Breneman, K. Kersten, P. Schroeder

**Affiliations:** 1 Department of Earth, Planetary and Space Sciences, and Institute of Geophysics and Planetary Physics, University of California, Los Angeles, USA; 2 Space Sciences Laboratory, University of California, Berkeley, USA; 3 NASA Goddard Space Flight Center, Greenbelt, MD USA; 4 Institute for Space-Earth Environmental Research, Nagoya University, Nagoya, Japan; 5 Kanazawa University, Kanazawa, Japan; 6 Tohoku University, 6-3, Aoba, Aramaki, Aoba Sendai, 980-8578 Japan; 7 Institute of Space and Astronautical Science, Japan Aerospace Exploration Agency, Sagamihara, Japan; 8 Korea Astronomy and Space Science Institute, Daejeon, South Korea; 9 Cottage Systems, Iowa City, IA USA; 10 Space Sciences Department, The Aerospace Corporation, Chantilly, VA USA; 11 Department of Earth and Space Sciences, University of Washington, Seattle, WA USA; 12 Department of Physics and Astronomy, Dartmouth College, Hanover, NH USA; 13 Department of Physics, Montana State University, Bozeman, MT USA; 14 Santa Cruz Institute of Particle Physics and Department of Physics, University of California, Santa Cruz, CA 95064 USA; 15 European Space Agency, ESAC, SCI-OPD, Madrid, Spain; 16 ADNET Systems Inc., NASA Goddard Space Flight Center, Greenbelt, MD USA; 17 Institute of Space and Plasma Sciences, National Cheng Kung University, Tainan, Taiwan; 18 Academia Sinica Institute of Astronomy and Astrophysics, Taipei, Taiwan; 19 Department of Earth and Planetary Science, Graduate School of Science, University of Tokyo, Tokyo, Japan; 20 Graduate Institute of Space Science, National Central University, Taoyuan, Taiwan; 21 National Centers for Environmental Information, National Oceanic and Atmospheric Administration, Boulder, CO USA; 22 Cooperative Institute for Research in Environmental Sciences (CIRES) at University of Colorado at Boulder, Boulder, CO USA; 23 Space Weather Prediction Center, National Oceanic and Atmospheric Administration, Boulder, CO USA; 24 The Johns Hopkins University Applied Physics Laboratory, Laurel, MD USA; 25 International Center for Space Weather Science and Education, Kyushu University, Fukuoka, Japan; 26 World Data Center for Geomagnetism, Kyoto Data Analysis Center for Geomagnetism and Space Magnetism, Kyoto University, Kyoto, Japan; 27 National Institute of Polar Research, Tokyo, Japan; 28 Hida Observatory, Kyoto University, Kyoto, Japan; 29 Laboratory for Atmospheric and Space Physics, University of Colorado, Boulder, CO USA; 30 Department of Physics and Astronomy, University of Iowa, Iowa City, IA USA; 31 Department of Geophysics, Kyoto University, Kyoto, Japan; 32 Physics Department and Space Science Center, University of New Hampshire, Durham, NH USA; 33 Southwest Research Institute, San Antonio, TX USA; 34 National Center for Atmospheric Research, Boulder, CO USA; 35 Laboratoire de Physique des Plasmas, CNRS/Ecole Polytechnique/Sorbonne Université/Univ. Paris Sud/Observatoire de Paris, Paris, France; 36 Center for Space Physics, Department of Mechanical Engineering, Boston University, Boston, MA USA; 37 Institute for Geophysics and Extraterrestrial Physics, Technical University of Braunschweig, Braunschweig, Germany; 38 University of Calgary, Calgary, Ontario Canada; 39 Center for Space Physics and Department of Electrical and Computer Engineering, Boston University, Boston, MA USA; 40 Space Research Institute, Austrian Academy of Sciences, Institute of Physics, University of Graz, Graz, Austria; 41 The Aerospace Corporation, El Segundo, CA USA; 42 University of Minnesota, Minneapolis, MN USA

**Keywords:** Space plasmas, Magnetospheric physics, Planetary magnetospheres, Solar wind, Ionospheric physics, Geospace science

## Abstract

With the advent of the Heliophysics/Geospace System Observatory (H/GSO), a complement of multi-spacecraft missions and ground-based observatories to study the space environment, data retrieval, analysis, and visualization of space physics data can be daunting. The Space Physics Environment Data Analysis System (SPEDAS), a grass-roots software development platform (www.spedas.org), is now officially supported by NASA Heliophysics as part of its data environment infrastructure. It serves more than a dozen space missions and ground observatories and can integrate the full complement of past and upcoming space physics missions with minimal resources, following clear, simple, and well-proven guidelines. Free, modular and configurable to the needs of individual missions, it works in both command-line (ideal for experienced users) and Graphical User Interface (GUI) mode (reducing the learning curve for first-time users). Both options have “crib-sheets,” user-command sequences in ASCII format that can facilitate record-and-repeat actions, especially for complex operations and plotting. Crib-sheets enhance scientific interactions, as users can move rapidly and accurately from exchanges of technical information on data processing to efficient discussions regarding data interpretation and science. SPEDAS can readily query and ingest all International Solar Terrestrial Physics (ISTP)-compatible products from the Space Physics Data Facility (SPDF), enabling access to a vast collection of historic and current mission data. The planned incorporation of Heliophysics Application Programmer’s Interface (HAPI) standards will facilitate data ingestion from distributed datasets that adhere to these standards. Although SPEDAS is currently Interactive Data Language (IDL)-based (and interfaces to Java-based tools such as Autoplot), efforts are under-way to expand it further to work with python (first as an interface tool and potentially even receiving an under-the-hood replacement). We review the SPEDAS development history, goals, and current implementation. We explain its “modes of use” with examples geared for users and outline its technical implementation and requirements with software developers in mind. We also describe SPEDAS personnel and software management, interfaces with other organizations, resources and support structure available to the community, and future development plans.

**Electronic Supplementary Material:**

The online version of this article (10.1007/s11214-018-0576-4) contains supplementary material, which is available to authorized users.

## Introduction

Since the early days of space research, space missions have been comprised of multi-instrument investigations producing particles and fields data products. Analysis has relied on correlative studies of such in-situ measurements along with auroral images, ground-based magnetograms, ionospheric precipitation data or radar one- or two-dimensional flow fields. A relatively small set of data product types has been produced by a set of space physics instruments that have seen incremental improvements over many decades. Traditionally, analysis tools have been home-grown, at the hardware development institution, and have been modified or upgraded from one mission to the next to follow computer and language evolutionary trends (aiming to preserve legacy code at the expense of flexibility and generality). This is not surprising, given the technical and programmatic difficulties involved in exchanging and interpreting data produced at different institutions. In fact, not too long ago, several of us had to physically visit instrument home institutions to obtain the native data in their most useful, archival form: microfiche, print or 9 inch track tapes.

Things have changed significantly since then. First, data repositories are now digitally accessible quickly and openly, through data repositories (e.g., NASA’s Space Physics Data Facility, SPDF, or NOAA’s National Centers for Environmental Information, NCEI) or distributed databases (e.g., Virtual Observatories, VxOs). Metadata (data descriptors) facilitating queries and on-line documentation are widely available for users to interpret these data. This transformation was precipitated in great part due to NASA’s implementation of International Solar Terrestrial Physics (ISTP, Williams 1980; Russell 1995; https://www-istp.gsfc.nasa.gov/) program guidelines for data storage in a common data format (CDF) (see: https://cdf.gsfc.nasa.gov/) in the 1990’s, and in part due to a Heliophysics science data management policy, version 1.0, released in 2007, requiring missions to provide to the user community access methods to scientifically useful data and analysis tools (for latest version and change-log see: https://hpde.gsfc.nasa.gov/Heliophysics_Data_Policy_v1.2_2016Oct04.html). Second, a number of missions with multiple spacecraft (e.g., the National Oceanic and Atmospheric Administration, NOAA’s GOES series, see: https://www.ngdc.noaa.gov/stp/satellite/goes/; the European Space Agency’s Cluster mission [Escoubet et al. 2001], NASA’s THEMIS [Angelopoulos 2008], Van Allen Probes [Fox and Burch 2013], and Magnetospheric Multiscale, MMS [Burch et al. 2016] missions) and similar instrumentation are beginning to work synergistically. These space missions also rely on ground-based assets, such as the THEMIS ground-based observatories (Mende et al. 2008), the Ultra Large Terrestrial International Magnetic Array, ULTIMA (Yumoto et al. 2012), and the Super Dual Auroral Radar Network, SuperDARN (Greenwald et al. 1995). Ground and space assets together reveal global magnetospheric connections between processes that extend from regional scales (1–3 Earth radii, i.e., a small fraction of magnetospheric regions, such as cross-tail width, dayside extent, etc.) to kinetic scales (electron or ion gyroradius or inertial lengths). The emergent H/GSO (optimized through the utilization of THEMIS’s fuel that allows optimal phasing of the THEMIS orbits with MMS orbits and ground-based observatories) has been endorsed as a concept by the Heliophysics Decadal Survey [National Research Council 2013] as part of its DRIVE initiative. This trend of increasing, distributed databases from multiple missions is expected to continue as Constellation Class missions [Angelopoulos and Panetta 1998; Siscoe 1998] come closer to reality and ground-based assets increase in number, data volume and complexity.

Increasing the science return gained from common data storage guidelines and an open-data environment is highly desirable to capitalize on the programmatic changes at NASA’s Heliophysics division regarding data access. At a time of an exponential increase in the number of space physics missions and ground-based assets, which need to work tightly together towards an efficient H/GSO, the duplicative effort of querying, acquiring, ingesting into a common analysis platform and visualizing time series data, including images, becomes disproportionately large compared to the time available for analysis and interpretation. Tools for efficient utilization and scientific exploration of such datasets are therefore highly desirable to increase the scientific return in this golden era of space research.

The need for standardized ingestion and analysis tools to work across disparate space physics datasets has existed for decades but no comprehensive and robust solution had emerged until recently. This was not due to lack of trying. Some solutions offer platform-independent visualization, e.g., Java-based Autoplot (http://autoplot.org, Faden et al. 2010); and python-based SpacePy (https://pythonhosted.org/SpacePy/, Morley et al. 2010). However, analysis under those packages is limited to what an instrument team prescribes via the data products it distributes. Various solutions have stemmed from specific projects or individuals, e.g., the Southwest Data Display and Analysis System, SDDAS (http://www.sddas.org/); the University of California Berkeley Space Sciences Laboratory’s Science Display Tool, SDT (Pfaff et al. 2001; McFadden et al. 2001); the University of California Los Angeles Institute of Geophysics and Planetary Physics’ UNIX-based Data Flow System, DFS, and Windows-based Space Physics Line-plotting and Analysis Shell, SPLASH (http://release.igpp.ucla.edu/igpp/splash, Wolkovitch et al. 1997); and the Imperial College London’s and Queen Mary and Westfield College, University of London’s Science Analysis System, QSAS (http://www.sp.ph.ic.ac.uk/csc-web/QSAS/, Allen 2010). These solutions were either restricted to one operating system, are written in a low-level language that requires significant maintenance support as operating systems evolve, or both. Generic analysis tools based on platform independent, commercial software (Excel, IDL, MATLAB) are either not standardized yet for space physics applications or are quite expensive (e.g., AVS, http://avs.com), or both. Such generic packages are also vulnerable to the fast pace of change in hardware and software technologies. Robust, flexible, and easy to use and maintain, multi-mission, multi-instrument tools for space physics data analysis have been previously absent.

A recent noteworthy exception, which evolved from Lockheed Martin’s long-term involvement in solar imaging is SolarSoft, or *SolarSoftWare*, SSW (http://www.lmsal.com/solarsoft). This IDL-based (platform independent) library of routines, geared primarily towards solar imaging and Flexible Image Transport System (FITS) files (https://fits.gsfc.nasa.gov), facilitates coordinated data analysis from multiple (like) instruments and provides an evolutionary, expandable environment for integrating other types of instruments and ancillary data bases. It was developed since the time of the Yohkoh mission, launched in 1991 (Ogawara et al. 1991) thanks to the commonality in data formatting of solar and astrophysical images enabled by FITS files at NASA/GSFC, that had been standardized 10 years prior, though it is not limited to such files. While the SSW concept is also ideal for in-situ measurements in space physics, the SSW libraries were mostly developed with imaging in mind.

A similar evolutionary path to SSW has recently emerged in the space physics community and is the basis of this paper. Thanks to the aforementioned standardization of data file formats through CDFs in the ISTP era and the need for comprehensive multi-instrument data analysis on the FAST mission at the Space Sciences Laboratory (SSL), a set of libraries that introduced data in a common, IDL-based environment for joint analysis was developed to address the needs of that mission in the early 1990’s as an alternate to SDT (McFadden et al. 2001). These IDL libraries also allowed commonality in analysis for similar particles and fields instrumentation produced at SSL for other missions, e.g., on WIND (Lin et al. 1995) and Cluster (Rème et al. 1997). With the advent of the THEMIS mission, which structured its data analysis environment to benefit from these packages starting from its first proposal in 1998 (Delory et al. 1998) and implemented during its science analysis planning phase starting in 2003 (Angelopoulos 2008), a general-purpose analysis system took shape independent of, parallel to, and using the same design principles as SSW. The THEMIS Data Analysis System (TDAS) relied heavily on legacy software but introduced (through a critical mass of software engineers) a regimen of quality assurance, suite-testing, version control, and backwards compatibility testing (subject to CDF and IDL version constraints), strong ties with SPDF, and community support. TDAS was designed to incorporate ground-based observatory images through CDFs and be expandable to ancillary datasets that were useful to enhance THEMIS’s system-science objectives (e.g., more than 100 ground-based observatories, geomagnetic indices, NOAA GOES, WIND, ACE, etc.). By the launch of the THEMIS mission in 2007 FITS files had also been incorporated in TDAS for better interface with SSW. Soon thereafter other projects (NASA’s Van Allen Probes; the Japan Aerospace Exploration Agency’s Energization and Radiation in Geospace, ERG [Miyoshi et al. 2018a, 2018b]; the Japanese inter-university upper atmosphere global observation network project IUGONET (Hayashi et al. 2013; http://www.iugonet.org); SuperDARN; NASA’s Mars Atmosphere and Volatile Evolution mission, Maven [Jakosky et al. 2015]) started utilizing it to conduct or prepare for their own multi-instrument, multi-mission data analysis.

Now, a decade after the first TDAS operational implementation, community endorsement of its design principles has led to the design of a software development superstructure, SPEDAS, a generalized software development platform, with distributed software developing teams and a core team of developers coordinating mission or instrument “plug-ins” based on a set of guidelines. Each plug-in is a separate branch of SPEDAS, a system of data query and retrieval codes integrated with standardized, generic visualization and analysis software at the core of the SPEDAS library. SPEDAS also incorporates a Graphical User Interface (GUI) that enables novice users to quickly introduce, analyze and plot data without the need for a paid IDL license. Individual missions create and support plug-ins (like THEMIS’s TDAS, IUGONET’s data analysis system UDAS, BARREL’s data analysis system BDAS [Woodger et al. 2015], MAVEN’s data analysis system MDAS, and ERG’s analysis software [Hori et al. 2013, 2015]). SPEDAS lead developers ensure core library stability and plug-in compatibility and inter-operability. The SPEDAS team has successfully demonstrated several models of use (ranging from zero, to minimal, to full IDL license cost) according to the financial means, network speed and institutional support of a specific user type/group (be they a mission, a student organization, a professional society or other). This flexibility tailors the installation to the user, and therefore ensures the widest possible utilization of SPEDAS by the community, maximizing the science return from Heliophysics’ missions.

In late 2017 NASA HQ provided SPEDAS modest funds to ensure continued community support of legacy missions (as well as of ancillary non-NASA missions critical for the emerging H/GSO), while the Ionospheric Connection Explorer (ICON, Immel et al. 2018) and Parker Solar Probe (PSP, Fox et al. 2016) have also started to develop their own plug-ins in anticipation of launch. Recent collaborations with the SPDF have led to successful ingestion of all ISTP-compatible SPDF data into the same software system as proof of concept of an emergent powerful new analysis platform. Thus, at this stage SPEDAS represents a grass-roots software development platform that requires only modest resources to maintain thanks to the in-kind contributions from dozens of programmers across inter-agency and international missions and ground-based observatories. SPEDAS was developed on the premise of platform independence. Therefore, as software technologies evolve, options for SPEDAS’s future evolution to keep up with the trends have to be thoughtfully considered. In that regard, it should be emphasized that the specific software implementation (presently using IDL) was based on considerations of code heritage, software development and maintenance cost, robustness, expert support staff availability, and other technical considerations. The SPEDAS structure, however, is transferable to other machine-independent languages or architectures. SPEDAS is thus expected to adjust as external (industry) conditions change and the relative weight of those considerations evolve with time.

In this paper we introduce SPEDAS to the user and software developer communities. In Sect. 2 we overview SPEDAS’ design philosophy and goals, and in Sect. 3 we discuss its historical evolution that led to many of its design choices. Readers interested in learning quickly what SPEDAS is, how it can be used for analysis or how it can serve their project’s needs (e.g., with a plug-in) can bypass these two sections. Section 4, intended for the user community, outlines the SPEDAS top-level capabilities in various modes of use. It is a self-sufficient introduction to SPEDAS with references to more extended documentation, tutorials, YouTube videos and on-line recorded webinars. Section 5, intended for developers, explains the SPEDAS architecture, expandability, flexibility, data access control, “plug-in” concept implementation details, the division line between project (“plug-in”) team and SPEDAS (core) team support and the personnel and software management structure and practices. Section 6 outlines on-going efforts for SPEDAS expansion during the next few years, notably interfacing with SPDF, Autoplot and the staged-development of a Python version of SPEDAS, PySPEDAS. Section 7 discusses potential expansions to include HAPI, planetary data and numerical modeling. Section 8 concludes with comments regarding SPEDAS’ future role in Heliophysics.

## Design Philosophy

By virtue of its inheritance from TDAS, SPEDAS was designed to be a machine-independent, easy-to-use data analysis system and software development environment, suitable for all space physics missions. It takes advantage of significant infrastructure already in place from two decades of development on several NASA missions. It puts in place a grass-roots software development platform for user support. This entails a core team providing developers’ guidelines, developer and user support, software quality assurance, test suites, and regression testing. By organizing and unifying the already significant but distributed H/GSO’s software development efforts and by optimally serving the user community, it creates an efficiently operating general-purpose data analysis environment. SPEDAS can be used in two mutually consistent modes: a command-line mode and GUI mode. A third mode, web-services, can be built upon either of the previous modes but relies on a remote server that runs IDL and presents the results on the web.

SPEDAS allows the user to (1) find the relevant data from the vast inventory of missions and data repositories without the use of a separate web browser or separate download operations, (2) ingest the data from heterogeneous sources with different native formats and resolutions into a universal data structure, (3) carry out a wide range of data analysis operations in a single, user-friendly platform, and (4) display the data in publication quality format. It works by empowering the mission science and software experts to develop and share their mission-specific data ingestion (and even calibration) tools, in return for benefiting from processing and visualization tools common to most space physics missions. The command-line mode is suitable for the more experienced data analysts and supports powerful crib-sheets (command sequences which can be simple or quite involved), dozens of examples to get started with, learn and copy from, or modify, and on-line documentation. It enables the most powerful and versatile data analysis experience possible. The GUI mode allows users to carry out most operations intuitively (although supporting documentation is available), and with little typing. The SPEDAS user-friendly, developer-friendly environment is not achieved at the expense of efficiency or adaptability. Researchers can copy and revise existing routines in this open source system to satisfy their specific needs (if not already met), or even develop and “check-in” new routines in the distribution. Under www.spedas.org, sharing of newly developed tools is encouraged and facilitated by a wiki page and mail-lists, and a process exists for integrating those tools into the SPEDAS software distribution for public usage and subsequently informing the community about recent software updates. This minimizes unforeseen duplication of efforts by like-minded scientists and promotes an evolutionary environment going beyond data analysis systems developed and distributed in the past.

The SPEDAS system was also designed to facilitate mission-implementation of the overarching principles of the aforementioned NASA Heliophysics Science Data Management Policy, in particular the requirement for easy electronic access and appropriate analysis tools. These requirements are levied on missions according to the “Rules of the Road” specified in the above policy document. The Principal Investigators (PIs) are required to provide “access methods … and analysis tools equivalent to the level that the PI uses”. This policy document also imposes a NASA Heliophysics “top-down” vision across all missions, to promote effective software analysis tools by contributing to the Heliophysics Data Environment, and notes that SolarSoft is one of those tools already available to the community. However, the responsibility for developing a common data analysis platform across all space physics missions was not delineated at the time of the latest version of the above policy document, as it was unclear then what vehicle(s) could fulfill that need. Thanks to the successful implementation of TDAS and the in-kind contributions to the community through the growth of SPEDAS in 2015–2017, this has changed. In September 2017, through a NASA/HQ contract to UCLA this responsibility was placed on the SPEDAS core development team through modest funding that enabled previous THEMIS human resources to be redirected towards non-THEMIS, community projects, such as currently operated H/GSO missions, legacy NASA missions, and ancillary space- and ground-based platforms. The NASA-funded SPEDAS efforts are geared towards: (1) SPEDAS software general maintenance, user community and developer-community support; (2) individual mission support in developing, launching and maintaining their “plug-ins”; (3) ensuring that checked-in software by the community is de-conflicted, versioned, backwards-compatible, quality-assured and consistent with the latest IDL versions (by interfacing with the software company, Harris Geospatial Solutions); (4) researching strategies for wider community inclusion, and (5) developing immunity towards industry evolution in software technologies (e.g., the emergence of Python).

SPEDAS was intended to support an efficient implementation of the H/GSO, in accordance with the intent of the aforementioned policy document and the recommendation of the National Academy’s Heliophysics decadal survey to optimize Heliophysics/Geospace resources across missions from multiple agencies, and NSF’s Geospace Environment Modeling, GEM, program (Wiltberger 2015). Towards that goal, SPEDAS was designed to help support multi-mission, multi-instrument analysis by facilitating searching, downloading, and ingesting scientific datasets from space physics missions via their own mission data portals, mirror sites, the Space Physics Data Facility (SPDF), Virtual Observatories (VxOs) and/or NASA data centers, and by providing a number of visualization and analysis tools to take full advantage of the archived and currently streaming data. Scientific exchanges are also facilitated by the exchange of short, high-level analysis code (crib-sheets) that can faithfully reproduce processing and the resultant plots and reveal the exact operations performed on the data, resulting in accurate interpretations and easy reproducibility. The latter, in fact, addresses a wider problem in all the sciences [Baker 2016]; this realization is slowly transforming our approaches to and expectations of published data and methods [Fanelli 2018]. SPEDAS is thus also intended to provide effective communication (via publications or other exchanges) of the detailed processing steps taken to generate the evidence supporting hypotheses. This allows scientific discussions to be devoid of misunderstanding, to transcend data manipulation and plotting, and to quickly jump into phenomenology, interpretation and data comparisons with models or theory.

As a result of these historical, policy, contractual and cultural reasons the following are the design principles upon which SPEDAS is currently based.

(A) *Platform independence*. To serve the widest segment of the community, SPEDAS must operate seamlessly under most major operating systems. To achieve this, it is most efficient to use a higher-level language that is well-supported either by a commercial entity or a grass-roots software development organization, in order to not have to re-invent this aspect of the SPEDAS system. Additionally, the data needs to be binary (for efficient packing) and easy to interpret and transfer across platforms—the CDF model works well, though other binary formats are also acceptable as long as intro-routines account for different binary representations at different architectures.
(B) *Project independence*. To serve an arbitrary space mission or ground-based observatory, SPEDAS must be project-agnostic. This is achieved by a decentralized set of routines which create a software development environment for any project to “plug-in” and work seamlessly with others. The code must be cleanly separated into project-unique and general-purpose libraries.
(C) *Availability of command-line, GUI and freeware options*. Users of varying degrees of sophistication and familiarity with the software need to be served. The GUI and freeware options allow novice users to get positive reinforcement with negligible initial investment that will encourage sustained use, growth of the user base and pro-active user time investment in the learning process. If the analysis tools prove their worth through use, and full SPEDAS capability is deemed necessary, paid options can be considered by the user.
(D) *Modularity of code*. Routines need to be separable into data introduction (query and data ingestion) routines, generic data analysis routines, and generic plotting and data export routines, in order to allow flexibility in contributions from projects, developers and the user community. This ensures that a distributed team of software developers, all experts in their own areas, are able to contribute efficiently to the SPEDAS project, rendering it most effective for the science community.
(E) *Community Support*. It is critical that good documentation, user training, new plug-in or analysis code development guidelines, a “help-line” for users, code maintenance and quality assurance are present. This allows SPEDAS to remain stable, robust and flexible.

The current system implementation adheres to the above design principles and reflects the best choices that were made at a given instant in time. The SPEDAS design choices do not represent hard system requirements and the team is constantly reassessing them to ensure the design remains optimal given the finite resources and time available. As software technologies change the system may require modifications, links to different packages or altogether revamping. In particular as new missions come to fruition, with more resources or needs, they are expected to take on and tackle major upgrades while their human capital may become the new core developer team. This flexibility in implementation will ensure SPEDAS remains useful in the long run for the Heliophysics community. The implementation details may change but the overarching principles, the design philosophy, is expected to remain. In the following section we describe the SPEDAS development history. This will help explain the current system design, which will be discussed in the follow-on sections.

## Development History

In the mid-1980’s it was realized that arriving at a comprehensive understanding of how energy flows through Earth’s space plasma environment requires multipoint observations. The International Solar Terrestrial Program created a multi-mission fleet to study various parts of the Sun-Earth system simultaneously and understand, through correlative analysis of the data, the modes and physics of the mass, momentum and energy flow. This was accompanied by standardization of binary (compact) data formats, complete with time and descriptors, known as the Common Data Format standard (CDF), for which translators were designed for various languages, such as C and Fortran, but were also incorporated in widely used commercial packages such as IDL and MATLAB. The ISTP-compatible flavor of that format became the standard means of (the required) data delivery of ISTP missions to the National Space Science Data Center (NSSDC) and its near-real time data repository, SPDF. This data format standardization for multi-spacecraft, multi-instrument missions became a major catalyst for standardization of data-ingestion routines, and enabled standardized analysis packages capable of such generic CDF interfaces to take off. As the ISTP data were being analyzed, it was also realized that higher-level languages, such as IDL and Matlab, offered the significant advantage of machine independence at the cost of software licenses, which was negligible compared to the personnel costs for ensuring a similar functionality in lower level languages, such as C or Fortran. Thus, by the early 90’s, when ISTP was in full swing, several groups around the country started developing IDL-based analysis packages that would ingest ISTP-compatible CDFs from various instruments and analyze data using standardized analysis routines. Those were typically instrument-team efforts, treating other instrument or other mission data as ancillary products. Ancillary products were ingested mainly through key-parameter datasets whose delivery was contractually required to SPDF during the ISTP era. One particular effort related to solar optical instruments that has blossomed into a generic, multi-instrument, multi-mission piece of software with quality-assurance provisions, came from the solar physics community, SolarSoftWare, as was discussed earlier.

Multi-instrument, multi-spacecraft space physics software for in-situ measurements naturally grew out of various team efforts utilizing higher-level language routines. UCLA’s DFS (a modular analysis package operating on flatfile binary files) was rewritten in IDL in 1994, and was introduced and used internally at JHU/APL by the lead author of this paper as a standalone analysis package for ISTP data analysis. The DFS IDL version was modular and relied on standardized “intro” routines for ISTP-compatible CDFs and internal representation of physical quantities using UCLA “flatfiles”. These structures contained data, “ctime” or Cline-time (seconds since 1966, the launch of the first UCLA magnetometer in space aboard the ATS1 satellite, named after a UCLA software engineer), data-descriptions and data-headers, in accordance with UCLA standards for representing binary time-series data in DFS.

Around the same time, a similar package was initiated by D. Larson at UCB’s Space Sciences Laboratory, to facilitate data analysis for the WIND mission. The internal quantity representation was IDL structures containing data, time (in seconds since 1970, otherwise known as Unix time, or posix time), and data limits, representing a physical quantity under the name “tplot” variable. Code was written for introducing, analyzing and plotting data from (electric and magnetic) fields instruments and (low and high energy) particle detectors. Standardized programs were used to manipulate these higher order quantities, and were general enough to apply to data from any satellite if they were cast in the same format. This formed the basis of the current SPEDAS program.

With the launch of the FAST mission, which was promoting efficient, concurrent multi-instrument data analysis under a PI-led program, the aforementioned, general purpose IDL-based code, presented an ideal solution for its data analysis needs. However, the low computing power personal computers of the time did not permit code in the high-level IDL programs to handle the expensive loading and calibration of the large FAST data files. Legacy code in the low-level language C was still critical for introducing FAST data into the user’s analysis environment. Thus, the early version of the FAST IDL-based package relied on binary data introduced into IDL using the C-based SDT as the interface, leaving only the ancillary key parameter data from ISTP missions and indices to be introduced by standardized “*intro*” routines. By the late ’90s it was apparent that although the initial FAST data analysis model was proposed and designed to primarily handle its data input and visualization through the C-based SDT, analysis was predominantly done at UCB and at affiliated institutions using the FAST IDL package, due to its platform-independence and low-maintenance requirements. However, as computers were becoming faster, different flavors of this IDL analysis package were transcribed for different instruments on SSL missions and proliferated quickly (WIND, Cluster, other). Conflicts in routine naming prohibited (or severely impeded) joint event analysis in the absence of a concerted effort to instill quality-assurance, the software remained limited to the confines of each individual mission, each cannibalizing code from the other.

The first implementation of platform-independent (in-situ) space physics software that adhered to most of the design elements of SPEDAS was developed as the THEMIS Data Analysis System (TDAS) in the early 2000’s. Using ISTP-compatible CDFs and routines that introduced Level-1 (uncalibrated, raw data) as well as L2 (calibrated data in physical units) it created an end-to-end solution for pre-launch instrument testing, post-launch on-the-fly data inspection and validation, and archival science data analysis. Additionally, given the THEMIS needs for all-sky imager (ASI) data access and analysis, CDFs for ASI images were also produced by the team (Sect. 8.1 in Mende et al. 2008) and were used for concurrent analysis with spacecraft data. The THEMIS program also needed to explore the connections across multiple spacecraft to study global phenomena (evidenced as substorms, dayside transients, nightside injections and inner magnetosphere particle energization). Thus, it also desired simultaneous measurements from many other national and international agency missions and ground networks. Therefore, THEMIS invested in the resources to establish multi-mission inter-operability, quality-assurance, version control and a distributed software development environment to accomplish its own primary and extended mission goals. By 2014, when TDAS V.9.0 was released, contributions had been made from more than two dozen programmers and scientists across more than a dozen institutions. At that time THEMIS was entering its third extended mission phase. ERG, IUGONET, NASA’s Balloon Array for Radiation-belt Relativistic Electron Losses (BARREL) mission [Millan et al. 2011] and MMS had already opted to use the same software system to create their own, full-fledged, multi-instrument analysis packages. Other projects concurrent to THEMIS and its lunar extension, ARTEMIS, were also introduced into the TDAS software distribution (e.g., Kaguya [Kato et al. 2010]) benefiting the emergent H/GSO. At that time it was realized that the potential of TDAS to ingest archived data from most legacy missions (going back to Apollo), currently on-going space missions and ground-based observatory datasets which were parts of the H/GSO, as well as missions which were then under development (ICON, PSP) was very powerful. In response to the growing requests for support of multiple missions the TDAS team initiated a code re-organization to create SPEDAS Version 1.0, in its present incarnation, in August 2014. SPEDAS, a software superstructure, incorporated THEMIS’s TDAS as a standard “plug-in” no different in priority or hierarchy than other missions. SPEDAS thus became an entity independent of THEMIS and its home institution. SPEDAS started to reside in http://spedas.org, and was operated as a grass-roots software development environment to serve the community of Heliophysics users with in-kind support from THEMIS and allied projects. While the initial responsibility of launching, organizing, maintaining, and supporting the greater community rested upon the initiating team, the expectation was that this responsibility could naturally evolve to other, more heavily endowed missions in the future. However, by 2016 the task of supporting other missions was already exceeding the professed role of the THEMIS mission for H/GSO coordination, especially in an extended phase. Luckily, in response to a NASA request for proposals for a Heliophysics “one-stop-shop” analysis service, allowing efficient use of multi-instrument, multi-spacecraft datasets, the SPEDAS team was selected to carry out these services for 5 years, starting in September 2017. At that point, SPEDAS was endorsed by NASA and the team started to pro-actively incorporate additional missions (Cluster, Geotail, …) and support extended services (link to Autoplot, development of Python solutions, link to Heliophysics API, etc.). The current paper describes the status of SPEDAS well into its first formal year of existence under NASA support, and four years after the initial concept was first launched.

## SPEDAS for Users

### What Is It?

SPEDAS is an open-source collection of high-level language routines that introduce, operate on, analyze, visualize, export and plot space physics data from multiple sources under a common, machine-independent environment. Platform independence is accomplished thanks to IDL’s multi-platform operation. It does not, however, come at the expense of development in other languages: legacy code in Fortran (e.g., for Tsyganenko models, Tsyganenko 2013, including field line mapping) as well as C, Python and other languages can be (and in fact is now being) compiled for multiple (major) platforms in advance of its distribution by the SPEDAS team, and the resultant machine-dependent executables, IDL dynamically loadable libraries or modules (.dll or .dlm files), can then be downloaded by the user specifically for their machine. Inevitable operating system upgrades are taken care of by IDL and its version upgrades, while the SPEDAS software is maintained by the core SPEDAS team, to ensure that no new bugs are introduced from new IDL versions, while maintaining (and documenting) backwards compatibility to the extent possible.

SPEDAS offers standardized access and import routines from distributed data repositories, written (at least for the highest resolution and highest quality mission data) by the individual mission teams (the experts). Multiple data processing levels and versions of the same dataset are allowed, if available at the remote site, with SPEDAS behavior controlled by standard keywords. Science processing and graphics are also standardized, containing a powerful set of legacy routines, good for all space physics datasets. SPEDAS is therefore a mission-independent analysis package, in the sense that any mission can “plug-and-play” in the same environment provided such data access routines are written. It is also decentralized as there is no single program to run it, or no single organization that is responsible for it. Rather it consists of a collection of guidelines, examples, legacy analysis and plotting code, maintained by a distributed community of users and developers. Structure and support is provided through modest NASA resources by the core team (which does not hold any rights to it), with in-kind contributions from project software teams.

### How Does It Work?

**Fig. 1:**
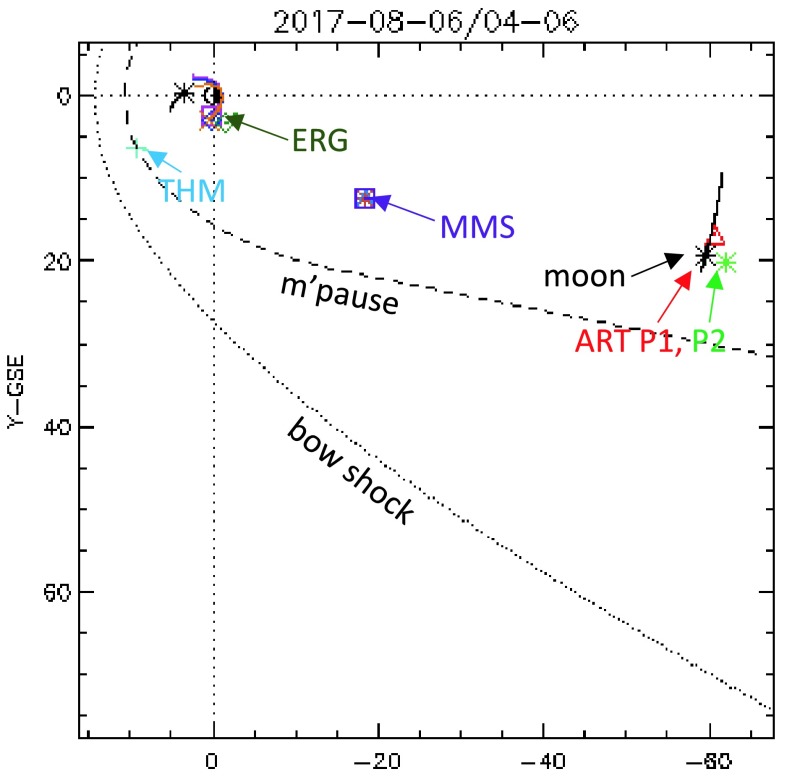
Spacecraft locations during a Heliophysics/Geospace System Observatory conjunction on 6 August, 2017, at 4–6 UT. THEMIS (TH-D) was at the magnetopause, ERG (Arase) at the pre-midnight inner magnetosphere, MMS in the near-Earth magnetotail and ARTEMIS in the magnetotail at lunar distances. (Modified from: http://themis.ssl.berkeley.edu click on Data → Summary Plots → Orbits: multi-mission)

**Fig. 2:**
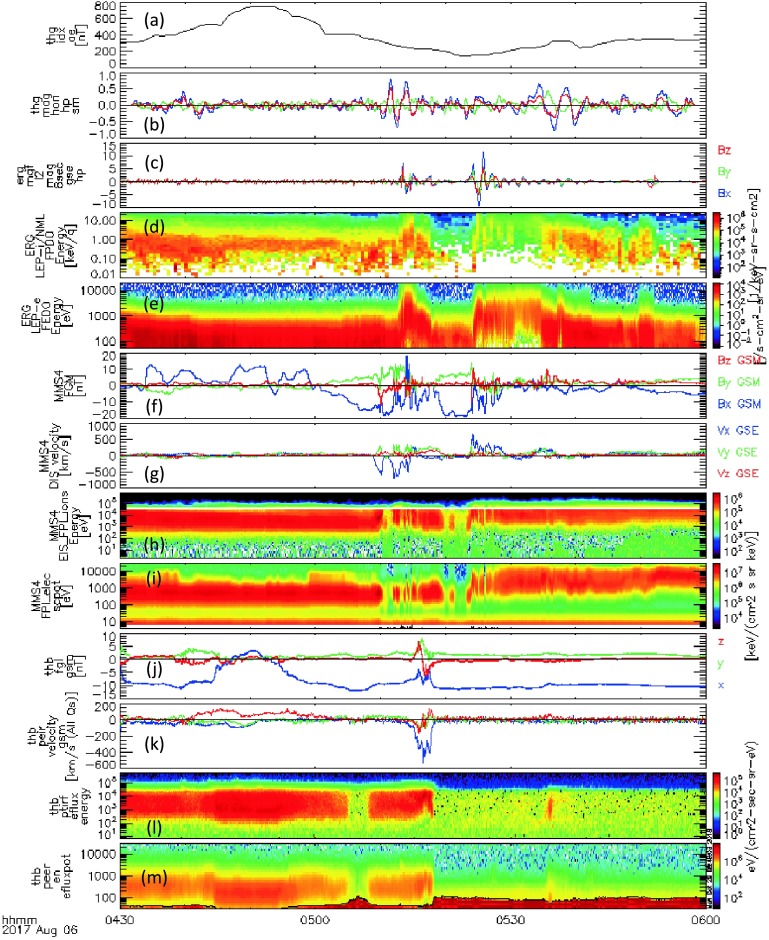
A few SPEDAS commands are sufficient to introduce, analyze and visualize H/GSO data from multiple instruments and spacecraft. (**a**) THEMIS PseudoAE index (unofficial, AE-like quantity derived from both the standard AE stations and additional stations in the American sector); (**b**) Pulsation data from mid-latitude magnetic station Honolulu indicating two activations; (**c**–**e**) ERG data showing magnetic field pulsations, ion injections, electron injections respectively; (**f**–**i**) MMS data showing negative-then-positive magnetic field $B_{z}$ component, negative-then-positive ion velocity $V_{x}$ component, ion spectra showing ion heating and electron spectra showing electron heating, respectively; (**j**–**m**) ARTEMIS data similar to MMS, showing a plasmoid (positive-then-negative $B_{z}$), moving tailward ($V_{x}<0$) and accompanied by ion and electron heating. This shows large-scale coupling in the magnetotail during this substorm

SPEDAS’s main modes of use are command-line and a graphical user interface. A third, less common but nonetheless powerful mode of use is web-services. Figure 1 shows an H/GSO coordinated satellite observation of a substorm injection, setting the stage for the data to be presented in subsequent figures. This satellite conjunction event is a good example for demonstrating the usefulness of SPEDAS in accessing high-quality (high-resolution and arbitrary level of processing) data from multiple missions. During this event the entire magnetosphere from the magnetopause (THEMIS or THM) to the mid-tail at lunar distances (ARTEMIS, or ART) responds within a few minutes. For convenience only the AE index, pulsations from a single ground station (in Honolulu), and a few panels from the ERG, MMS 4 and ARTEMIS P1 spacecraft are shown in Fig. 2. Data from more satellites for this event will be shown later.

#### SPEDAS Command-Line Mode of Use

Figure 2 has been constructed using the command-line mode. A few commands typed in the IDL Console window or assembled into and executed from a user program are sufficient to introduce such multi-instrument, multi-mission and ground-based observatory data, while demonstrating the large scale connections across space and ground for this event. For the purposes of Fig. 2, shown in file “Figure02.pro” in the Supplementary Materials is a text file which serves as an IDL script, or IDL program (in this case the former); the commands can be inserted (cut-and-paste one-by-one or in multiple lines) in the IDL command window for execution. Programs like it can be run as a script in its entirety with a single command, in this case: “Figure2” (or equivalently in the IDL window press buttons “Compile”, then “Run”). The latter choice should be used judiciously and is only warranted if the script has been completely debugged prior to execution. This method’s casual use should also be avoided because in the event of a user-error the code would have to be recompiled and rerun in its entirety which is far more time consuming than a line-by-line execution. The cut-and-paste option is often the most useful, as it also allows the user to insert additional commands to view or analyze the data mid-way, prior to deciding on the optimal products, limits and making the final plots. Because command files like these are often work-in-progress, the name “crib-sheets” is often used to describe them. They can be exchanged between scientists to describe explicitly their data processing. However, they can also be published in the literature (as in this paper) permitting the authors to demonstrate to the community how exactly the publicly accessible data were processed to arrive at a published plot and scientific arguments made. In that case they would be expected to execute completely without errors assuming the correct IDL and SPEDAS versions are used.

Once the data have been downloaded on the user’s local system (cache), the user can repeat or alter the analysis and recreate the plot even without a connection (or with internet turned off) which is good for continuing the analysis at a location with poor connectivity (e.g. on the plane or at the airport). If connected, however, and if a new version of the data does become available at the remote data repository, SPEDAS will recognize this remote update based on a comparison of the creation date of the files at the local versus the remote locations, and will automatically perform a fresh download (unless commanded otherwise), overwriting the old data in the user’s machine. The requirements for the command-line mode are a full IDL license, a copy of the SPEDAS software and an internet connection with reasonable speed for the volume of data desired.

The workhorse behind the SPEDAS operation is the concept of “tplot” variables. These are character strings, declared in double or single quotes, “ ” or ‘ ’, each pointing in memory to an IDL structure containing data arrays that represent a physical quantity. These are all transparent, though accessible to the user. Each IDL structure contains time, data (typically of 1 or 2 dimensions, though 3 or higher dimension data can be accommodated by using the command “reform” as in the case of energy, theta, phi angles plots for particle data), plotting ‘limits’ specified by the user, and default values for those limits (‘dlimits’) initially specified at the time the data is read. It may also contain substructures, themselves pointing to arrays. All these arrays referenced by the IDL structure are thus linked (tagged) together as tags of the IDL structures and substructures. The variables (along with their dlimits) are ordinarily introduced automatically by the load routines, e.g., the command: “$\mathit{mms\_load\_eis}, \mathit{probe}=[4]$” within Figure02.pro loads a number of such tplot variables from the energetic ion spectrometer (EIS) instrument [Mauk et al. 2016] on the MMS4 spacecraft. The time interval pertaining to the analysis session can be specified at the start of the session, affecting all subsequent calls and plots, with the command: “*timespan, ‘2017-08-06/04:30’, 1.5,/hours*”. The time range can be further adjusted (if needed) during the session independently for each variable and routine by the choice of a keyword (trange); as long as the data has been previously loaded they will be acted upon. The tplot variables that are present at any given point during the user’s current data analysis session can be listed by typing the command: “tplot_names”.

The user at this point can: view the raw data values introduced in any tplot variable by interrogating the underlying arrays in memory; modify the data and create new IDL arrays as needed; or create new tplot variables with the help of the existing ones by operating on them. This can be done with the set of commands: get_data and store_data. An example of a tplot variable in Fig. 2(h) and Figure02.pro is ‘mms4_epd_eis_extof_proton_flux_omni_spin’ containing omnidirectional proton differential energy flux from the EIS instrument on MMS4, as introduced previously. The data can be read out as arrays with the command: *get_data*, ‘*mms4_epd_eis_extof_proton_flux_omni_spin*’, $\mathit{data}=\mathit{myeis}$, $\mathit{dlim}=\mathit{myeisdlim}$, $\mathit{lim}=\mathit{myeislim}$ and the arrays thus created are tags in the IDL structure “myeis”: $\mbox{myeis.x} = \mbox{time}$ array, $\mbox{myeis.y} = \mbox{data}$ array, $\mbox{myeis.v} = \mbox{energy}$ array, whereas the limit values are contained in structures: $\mbox{myeisdlim} = \mbox{the}$ dlimit structure, and $\mbox{myeislim} = \mbox{the}$ limit structure. (The user can interrogate the latter structures using the commands: “*help, myeisdlim*,/*structure*” and “*help, myeislim*,/*structure*”.)

Extracting the arrays using the “get_data” call is not the most efficient way of manipulating the data, but it is a quick and fail-safe way to view the actual numerical values once they become thus available in array form. Products of user operation on these arrays can then be inserted compactly back into tplot variables with the command: “store_data”. An example of this is the creation and storage of a new quantity: ‘mms4_epd_eis_extof_proton_eflux_omni_spin’ from the number flux and energy values contained in the structure myeis, in the crib-sheet Figure02.pro.

The most efficient way of viewing, analyzing and processing the data is by using them directly in their tplot variable form. Higher-level commands in SPEDAS are used towards this goal. For example, the command: *“tplot, ‘mms4_epd_eis_extof_proton_flux_omni_spin’ ”* (here single quotes for the tplot variable are used on both sides but in the user’s command-line double quotes are also acceptable) will result in a single-panel plot without the user having to specify any limits or colors or other plot attributes (assuming the quantity’s dlimits were set properly upon loading by the load routine, if that was well-written by the instrument team). This is in fact a major advantage of the tplot system. It places the onus of the proper introduction and plotting of the variables on the instrument software team. The data is automatically recognized by SPEDAS as time-series, or spectra or images, and proper (default) dlimits allow immediate plotting with little user intervention. This facilitates data access and prevents miscommunications or errors in conveying the data-properties information to the user. Limits for tplot variables can be further specified or modified by users by the command “options”, as excerpted from Figure02.pro below:

$$\begin{aligned} &\textit{options},\ \textit{`mms4}\_\textit{fpi}\_\textit{eis}\_\textit{proton}\_\textit{omni}\_\textit{spin'},\ \textit{ylog}=1,\ \textit{yticks}=0,\ \textit{yminor}=10,\\ &\textit{yrange}=[2.,8.e5],\ \textit{ystyle}=1,\ \textit{zrange}=[3.e1,3.e6] \end{aligned}$$

Global (multi-panel) plot limits can be changed using “tplot_options”, for example in Figure02.pro: “*tplot_options, ‘xmargin’*, [$20,9$]”, sets the horizontal left and right plot margins in points. Note that most keywords are standard IDL plot keywords which can be found in the IDL help command (by design SPEDAS supports most IDL native keywords); non-standard ones are listed in the SPEDAS distribution (in crib_tplot.pro under general\examples which can also be opened into the editor by typing “.e crib_tplot” in the command line window).

In addition to plotting routines operating directly on tplot variables, other high-level routines can also operate on them, to perform data analysis. The natural sequence of operations for any single quantity of interest can be divided into Load, Analyze, Plot types of routines. Those operations are interwoven within a crib-sheet when multiple-data products even from a single instrument (let alone from multiple instruments or missions) are introduced. It did not make sense to impose any a priori code structure (as exists in DFS or AVS), and rather let the code be structured using organizational comments by the user. These comments are driven by the needs of the flow of scientific analysis. A partial list of common operations on tplot variables (besides just plotting) exists in crib_dproc.pro (“dproc” in this name simply denotes “data processing”) under general/examples in the SPEDAS software distribution (it can also be viewed by typing the command: “*.e crib_dproc.pro*”).

For example, in Figure02.pro the commands, *tsmooth2*, ‘*thg*_*mag*_*hon*’, 241 *dif*_*data*, ‘*thg*_*mag*_*hon*’, ‘*thg*_*mag*_*hon*_*sm*’, $\textit{newname}=\textit{`thg}\_\textit{mag}\_\textit{hon}\_\textit{hp'}$, $\textit{copy}\_\textit{dlimits}=1$ will first operate, with tsmooth2, on the one-dimensional (three-component vector) ground magnetometer data from Honolulu, with 3 s resolution (performing a 241 point running-average and producing the default-named variable: ‘thg_mag_hon_sm’) and then using dif_data they will subtract the latter tplot variable from the original one to create the high-pass filtered product. Notice no array extraction, operation, and tplot variable creation had to be initiated separately by the user, thus simplifying the code; such trivial get/store manipulations were completed internally. However, the user must be cognizant of the fact that the data points are assumed to have constant cadence and no gaps. If that is not the case, then degapping (using “tdegap” which inserts flags as not-a-number values, NaNs, when intervals longer than the prescribed time resolution are detected) and interpolation (using “tdeflag” to replace flags by a user-specified method, including linear interpolation) should precede this operation.

A very useful addition to the SPEDAS analysis tool-chest is the routine: “calc” which performs complex operations on tplot variables without having to “get” and “store” the data. An example of how the generic “calc” routine performs the identical (albeit trivial) operation as “dif_data” is shown in Figure02.pro following the “dif_data” call (it is commented out presently).

$$\begin{aligned} \textit{calc}, \textit{``}\,\textit{`thg}\_\textit{mag}\_\textit{hon}\_\textit{hp'} = \textit{`thg}\_\textit{mag}\_\textit{hon'}-\textit{`thg}\_\textit{mag}\_\textit{hon}\_\textit{sm'}\,\textit{''} \end{aligned}$$

Notice that the “calc” command internally parses the string that follows it in quotes (single or double quotes are both fine, as long as they are consistent with each other, and opposite to what’s used to declare the tplot variables within the expression). Arrays (denoted by un-quoted strings), variables and constants can be interleaved with tplot variables and operated upon. Examples of operators on such quantities within “calc” are: “∼ ++ −− − + ∗ / ^ < > $\&\&$ $||$ $\#$ $\#\#$ mod and”. Examples of functions supported are: “$\log(x[,\mbox{base}])$ $\ln(x)$ $\exp(x[,\mbox{base}])$ $\mbox{sqrt}(x)$ $\mbox{abs}(x)$ $\mbox{mean}(x,[,\mbox{dim}][,/\mbox{nan}])$ $\sin(x)$ $\arcsin(x)$ $\sinh(x)$ $\operatorname{arctanh}(x)$”. A full list can be found by executing the command: “*calc*, $\textit{operator}\_\textit{list}=o$, $\textit{function}\_\textit{list}=f$” and subsequently printing the output lists o and f (e.g., “*print*,*o*”). Information on this, and any of the SPEDAS routines is available both online at the SPEDAS wiki under Documentation (direct link here: http://themis.ssl.berkeley.edu/socware/spedas_3_1/idl/_spd_doc.html). Such information is also available as an html document in the user’s downloaded SPEDAS distribution files (top directory, called _spd_doc.html, and any subdirectories by subdirectory name) or by editing the subroutine corresponding to the command by typing “*.e mycommand*” for example “*.e calc*”. Many more examples of “calc” usage are provided in the routine’s header, viewable by typing “*.e calc.pro*” and also in crib_calc.pro in the examples directory, also accessible by typing “*.e crib_calc.pro*”. Both are also accessible with a standard text editor.

**Fig. 3:**
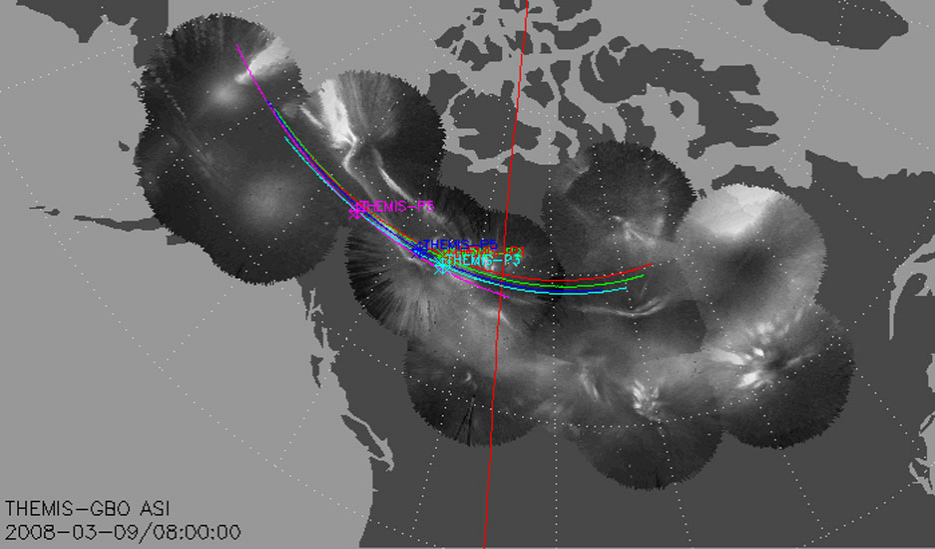
Mosaic of all-sky images obtained from THEMIS Ground-Based Observatories (GBOs) with mapped ground tracks of the THEMIS satellites over $\pm3~\mbox{hrs}$ around 08:00 UT, the time these images were taken and for which satellite locations are marked with an “X” next to the satellite identifier (P1–P5). The straight meridional red line marks magnetic midnight (dotted lines are constant magnetic latitude and longitude lines)

An example of plotting two-dimensional (2D) products (such as on-board produced frequency-time, or energy-time spectrograms, or images) with SPEDAS is shown in Fig. 3. In that figure image data from the all-sky cameras from the THEMIS Ground Based Observatory (GBO) array in CDF format are read and overlaid on a geographic map with superimposed geomagnetic coordinates and satellite projections (denoted by “X”) and ground tracks. The same principles as for vector variables apply in the ingestion and interpretation of 2D data, except the data tags of the tplot structures have an extra (second) dimension. A library of routines allowing specialized image projections to the same altitude, concatenation to remove overlaps, or suppress noise (moon, cloud or car light) and edge effects (trees, structures) is included in thm_crib_asi.pro under projects/themis/examples/basic in the SPEDAS distribution.

The SPEDAS installation can be found in http://spedas.org along with introductory videos, examples, recent SPEDAS presentations, currently supported missions, user’s guide, various crib-sheets (in example folders, often with subdirectories “basic”, “advanced” etc.) that are general to SPEDAS, mission-specific or instrument-specific.

#### SPEDAS GUI Mode of Use

The second main SPEDAS mode of use is the GUI. This does not necessitate a paid IDL license, but only a “Virtual Machine” IDL installation, which is free. This installation is bundled with the SPEDAS free distribution and the user need not download it from the IDL site separately (the SPEDAS team has paid the additional fees to be able to distribute it directly). The intent of the Virtual Machine is to allow third party software vendors to distribute IDL-based higher level applications (e.g., medical tomography) that can be run on client machines without the functionality of IDL in command-line. However, thanks to SPEDAS’s routine “calc”, which can operate on tplot variables, arrays and variables, after parsing, interpretation and internal redirection to IDL operations, the Virtual Machine accepts data analysis through “calc”. This allows the GUI to have nearly full functionality, to the extent that data operations can be done within “calc”. Data loading and plotting with the GUI have additional benefits over the command-line (as we discuss below) and therefore the GUI mode of use is a powerful alternative to the command-line, free of charge, and a complementary tool even for license-paying command-line users.

Except for data processing on tplot variables using “calc”, tplot usage is transparent to the GUI user (absent). This is because to represent the data the GUI does not use tplot variables internally, but rather one-dimensional data objects called “GUI variables”. Like tplot variables, these too are pointers in memory, so no data fetching or copying is needed to declare them once they are physically present there. The SPEDAS protocol is thus to introduce data even into the GUI mode first as tplot variables from the standard tplot variable load routines (behind the scenes). Once the data becomes accessible in memory the information it contains (data arrays, limits etc.) lends itself to GUI data object representation too, in parallel to tplot representation. Therefore, GUI and command-line modes are entirely compatible with each other, and can be used in parallel (an action on the data in one mode has an immediate effect on the data views through the other mode). Thus, the command-line user can take advantage of the additional GUI functionality, such as searching and introducing any ISTP-compatible SPDF dataset; easily navigating through and selecting many mission products without any familiarity with data location and their read routines; menu-driven data analysis, high-quality plotting and visualization capabilities, to name some.

An example of using the GUI to import, analyze and plot various quantities is shown next, in Figs. 4a–4c. It is for a time encompassing the same event as shown in Fig. 1, but provides information from NOAA’s Deep Space Climate Observatory, DSCOVR, mission at the L1 point (https://www.ngdc.noaa.gov/dscovr/), THEMIS-D (TH-D) at the magnetopause, and GOES 13 at geosynchronous very close to midnight ($\mbox{MLT}=23\mbox{--}24$). The AE index panel is repeated at the top for reference. The plot can be constructed immediately by importing a GUI “document” which contains the pre-recorded steps for creating the plot, as will be discussed later in this section. However, here we start by explaining what the GUI data loading, analysis and plotting steps look like, before we discuss saving and importing prior GUI sessions to recreate such plots.

**Fig. 4a:**
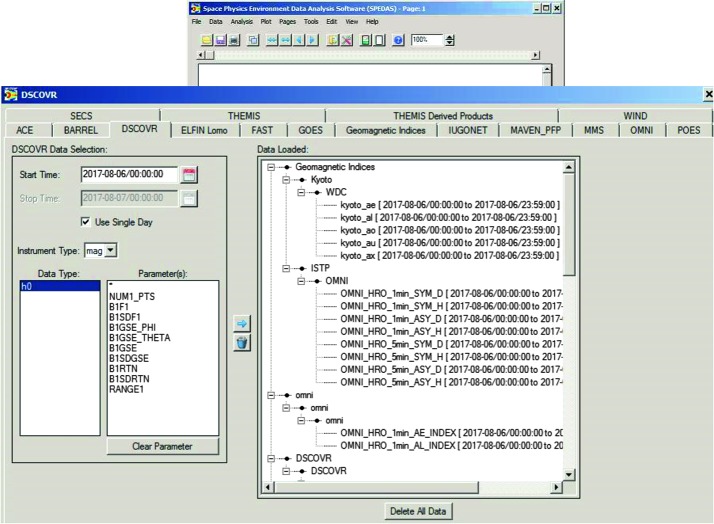
A representative Data Input session (the DSCOVR tab, invoked from “Data” → “Load Data using Plug-ins…” → “DSCOVR” tab) using the SPEDAS GUI with projects (missions, observatories, networks) each assigned its own tab. On the right is the list of all the data loaded at any point during the session (here Kyoto WDC-C data have been loaded, as well as OMNI data and DSCOVR data). The panel in the background is the main GUI panel, initially blank, but will be populated with multiple time-series panels once plotting occurs. It can also contain multiple plots (“Pages”) and the user can toggle between them from the appropriate menu button. The main menu contains all the GUI options including “Data” (for data input/output and switching between tplot and GUI variables), “Analysis” (which includes “Calculate…” and “Data Processing…”) and “Plot” (which includes “Plot/Layout Options…”)

Upon calling the GUI from the command-line as: “*spd_gui*” or “*spedas_gui*”, a blank panel (which will become the plot window) with a menu bar on top, appears. This is shown as the background panel in Fig. 4a. The user is then expected to Input some data from the data-loading routines and downlink services provided by SPEDAS plug-ins, or from other generic services (such as HAPI or SPDF’s Coordinated Data Analysis Workshop web, CDAWeb), or from previously downloaded CDF files residing at a directory in the user’s machine. These are the first four tabs under the main GUI menu item “Data”, and correspond to the choices: Data → Load Data from Plug-ins…, Data → Load Data using HAPI…, Data → Load Data using CDAWeb…, and Data → Load Single File. The first option is the most widely used and easiest for the user, as the user need not worry about fetching data files or locating the quantities to download. This invokes the “Load Data” pop-up window and is shown as the foreground in Fig. 4a. It contains a choice of tabs corresponding to missions, indices and ground-based observatories with relevant choices of parameters (instruments, spacecraft, data rate, data level, data type, etc.) and time (the default is the first plug-in in alphabetical order, and the default time is the date of the THEMIS first-results paper [Angelopoulos et al. 2009]). Initially, when no data have been loaded, the right panel appears blank. Once the user introduces, one-by-one, the data from the aforementioned missions through their respective tabs, the right panel is populated with the variable names corresponding to the loaded quantities. Figure 4a, foreground, shows the DSCOVR tab for the day (2017-08-06), Instrument Type (MAG), and Data type (h0) of interest selected. After clicking the right arrow in the middle of this panel these data are fetched from their remote location to the user’s data directory (on a personal computer the default is C:\data) and then introduced into the GUI. The data are introduced in “GUI variable” representation (internally broken down to 1D structures, though they are treated as bundled into quantities which are linked to the same time series as appropriate for their dimensionality, be they vector or spectral quantities) but this is transparent to the user. As mentioned earlier, there is a direct mapping from GUI to tplot variables; this can be enabled should the user desire to exercise it. This is done through the “Manage Tplot and GUI Variables” tab under the main menu item: “Data”. The same tab can be used to modify GUI variable attributes in the process of analysis (e.g., to fix errors or modify attributes that were incorrect or missing when the variables were first introduced from the mission data repository). The data can be then analyzed and plotted. Alternatively, data can be deleted or overwritten (if, for example, a larger or different timespan is desired), and analysis and plotting can proceed again.

Analysis can proceed either in GUI mode or in command-line mode (or both, interchangeably). Much of the GUI analysis can happen by operating on GUI variables, by activating them in the Data Processing window (in the main menu, under “Analyze” → “Data Processing”), then using one of the operations to the right. Here we will use as an example the very useful operation “Coordinate Transform” which can convert between different geophysical coordinate systems (GSM, GSE, SM, GEO, MAG, GEI, J2000). The coordinate system of the data to be transformed must have been declared internally in the “dlimits”, and if this did not happen upon the introduction of the data the user must correct it. At the time of writing this is the case with the DSCOVR B-field data which were introduced (as explained earlier in this section) in GSE coordinates but the load routines left the coordinate system undeclared during the introduction process, causing the coordinate transformation program to refuse the GSE-to-GSM rotation. To fix that the user can go to the “Data” → “Manage Tplot and GUI Variables” tab, select the variable “dsc_h0_mag_B1GSE”, manage it (use the “Manage a GUI Variable” button to change attributes) and declare its coordinate system to be GSE. Once that is done then the user can use the “Data Processing…” tab located under the “Analysis” top-level menu item, to first bring the GUI variable into the “Active Data” panel, select it there, and then click on the “Coordinate Transform” button the choice: GSM, which will create a new GUI variable with the name: “dsc_h0_mag_B1GSE_gsm” in GSM coordinates. Our next step is to create the total field for DSCOVR (it can be introduced too as a separate quantity, but computing it from the individual components allows us to demonstrate how to operate on the data). For that we will use “calc”. Under the “Analysis” top-level menu item, opening up the “Calculate…” tab (which brings up the panel shown in Fig. 4b), we write the “calc” operation in the scratch pad on the left. This should read (all in one line):

$$\begin{aligned} &\textit{``dsc}\_\textit{h0}\_\textit{mag}\_\textit{B1GSE}\_\textit{gsmt''} = \textit{sqrt}(\textit{``dsc}\_\textit{h0}\_\textit{mag}\_\textit{B1GSE}\_\textit{gsm}\_\textit{x''}\textasciicircum\textit{2}\\ &+\textit{``dsc}\_\textit{h0}\_\textit{mag}\_\textit{B1GSE}\_\textit{gsm}\_\textit{y''}\textasciicircum\textit{2} +\textit{``dsc}\_\textit{h0}\_\textit{mag}\_\textit{B1GSE}\_\textit{gsm}\_\textit{z''}\textasciicircum\textit{2}) \end{aligned}$$

Typing and editing variable names can be facilitated by selecting individual quantities on the right and pulling their names into the scratch-pad (clicking the left arrow does that). This avoids typos. When the operation is fully constructed and executed successfully (by clicking the “Run” button) a new GUI variable will be inserted into the list of available variables on the right (named: “dsc_h0_mag_B1GSE_gsmt”). The operation has been declared successful at the bottom left and the insertion of the new variable noted at the bottom right. If the operation fails, the failure mode will be also noted in that window. Note that functions and operators available are also shown on the right of the Calculate panel (and can be inserted into the scratch pad either by a click of the mouse or by direct editing).

**Fig. 4b:**
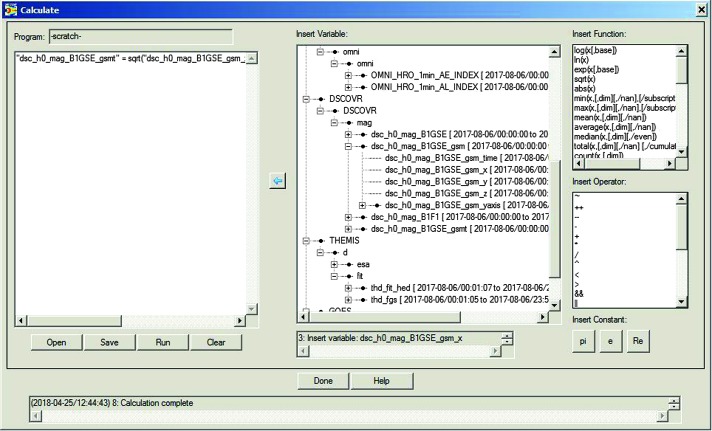
The “calc” routine can be used also in the GUI mode through the “Calculate” button, located under the “Analysis” main GUI menu item. It enables generic data analysis, including complex operations (one line per operation in the scratch pad on the left) well beyond what is possible by standardized packages available through the “Data Processing…” button (also located under the “Analysis” main GUI menu item)

Plotting the data using the functionality of the “Plot/Layout Options…” button (located under the top-menu button Plot in the GUI) is self-evident. Figure 4c shows the data from the aforementioned missions for this GUI session. The new variable we just computed, the total magnetic field from DSCOVR, is the fourth quantity we need to plot in the same (DSCOVR GSM XYZ magnetic field components) panel. It can be over-plotted along with its components by first unchecking the “Automatic Panels” button in the Plot → Plot/Layout Options… button, and then by inserting the total field in the same panel as XYZ.

**Fig. 4c:**
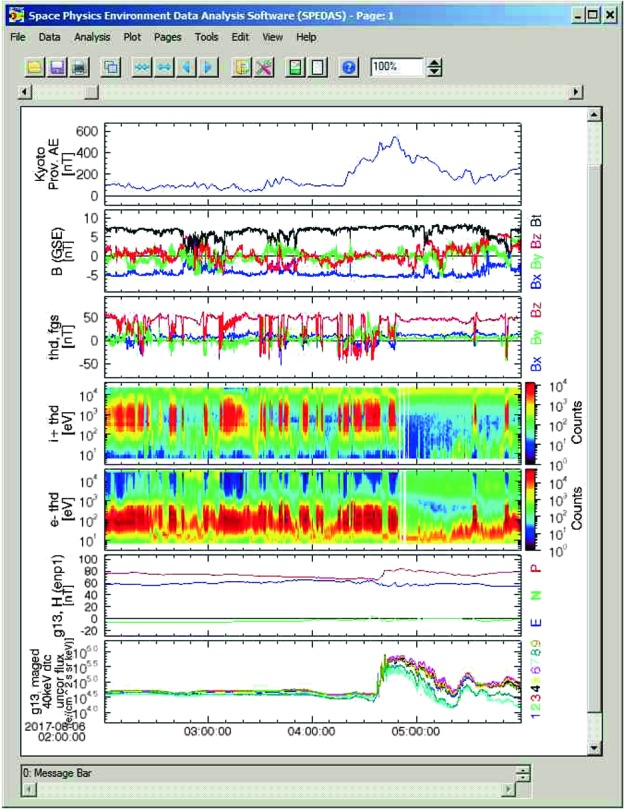
A representative multi-instrument, multi-mission plot from the GUI mode for a time encompassing that of Fig. 2. The AE index is shown on top, followed by the DSCOVR magnetic field (GSM coordinates plus total field), and the THEMIS-D magnetic field (GSM coordinates), ESA ion and ESA electron omnidirectional spectra. The bottom two panels are the GOES 13 magnetic field in ENP coordinates and the 40 keV electron energy flux from the 9 MAGED electron detectors (with different look directions). DSCOVR data have not been propagated from L1 to the subsolar magnetopause. Given the associated time delay of 50 min, it is evident that southward interplanetary magnetic field was responsible for the increased geomagnetic activity seen at the AE index between 04:20 and 05:40 UT. The GOES 13 electron flux enhancements corroborate that activity with injections seen at 04:40 and 05:20 UT. The latter pertains to the activity observed at ERG, MMS, and ARTEMIS seen in Fig. 2

Like the command-line mode, which uses crib sheets to save a clean set of the user’s actions for exchange and future reference, the GUI utilizes three types of files to achieve the same goal. These GUI files save useful analysis products and plots, such that they can be executed again, perhaps for different times or parameter settings. The first type of such files stores the set of “calc” operations done to the data—these can be saved into simple text files (e.g., “Figure04.calc.txt” in the Supplementary Materials). They can be modified with an editor external to IDL, and reloaded when needed (by clicking the button “Open” in the “Calculate” tab). Multiple lines in that file correspond to successive operations, and can result in highly sophisticated analysis. Multiple “…calc.txt” files can also be employed, corresponding to different user-actions on the data, at various stages of the analysis sequence using the GUI session. The second file type stores the user’s entire GUI command history session (loading data, loading templates or “calc” files, analyzing data, plotting) for later repeated use. These end-to-end sessions (most often leading to final plots for research or papers) are saved in files called GUI documents, suffixed.tgd (‘the GUI document’, e.g., Figure04.tgd). These can potentially get very large if operations are lengthy or circuitous, as in the case of exploratory analysis. Since there is no use in repeating aborted analysis paths, once the user is relatively confident in the sequence of operations needed to recreate a plot from scratch they can restart a fresh GUI session for recording a clean version of a well-scripted series of actions for future use, then save the.tgd file for future replay. This document file can either be modified with an editor to create repetitive incarnations of the sequence with a varying set of parameters or dates, or it can be used as a starting point for alternate repetitive operations to be implemented by the user from the GUI. The third file type stores plot templates (colors, labels, titles, etc.) in files suffixed.tgt (‘the gui template’, e.g., Figure04.tgt); they contain html code that fully specifies the plot/panel attributes.

With the combined use of the aforementioned three types of files (*.calc.txt; *.tgd; *.tgt) the GUI user has the same benefits as command-line users do from crib-sheets. These types of files being in American Standard Code for Information Interchange (ASCII) format, they can be easily exchanged, modified, referenced, parsed and patched. They can be used to successively build increased layers of sophistication, for example to adapt case studies of individual events on one satellite to multi-satellite, multi-case or statistical analyses. They not only enhance productivity of individual scientists, building confidence in their high-level processing, but also promote collaborations between scientists across institutions and time-zones, because they can communicate accurately and effectively the entirety of the coding involved in their discussions. This builds mutual trust in the analysis and permits elevated scientific discourse even in an era of numerous, voluminous datasets available at their highest cadence at distributed sites.

#### Web-Services Mode of Use

**Fig. 5:**
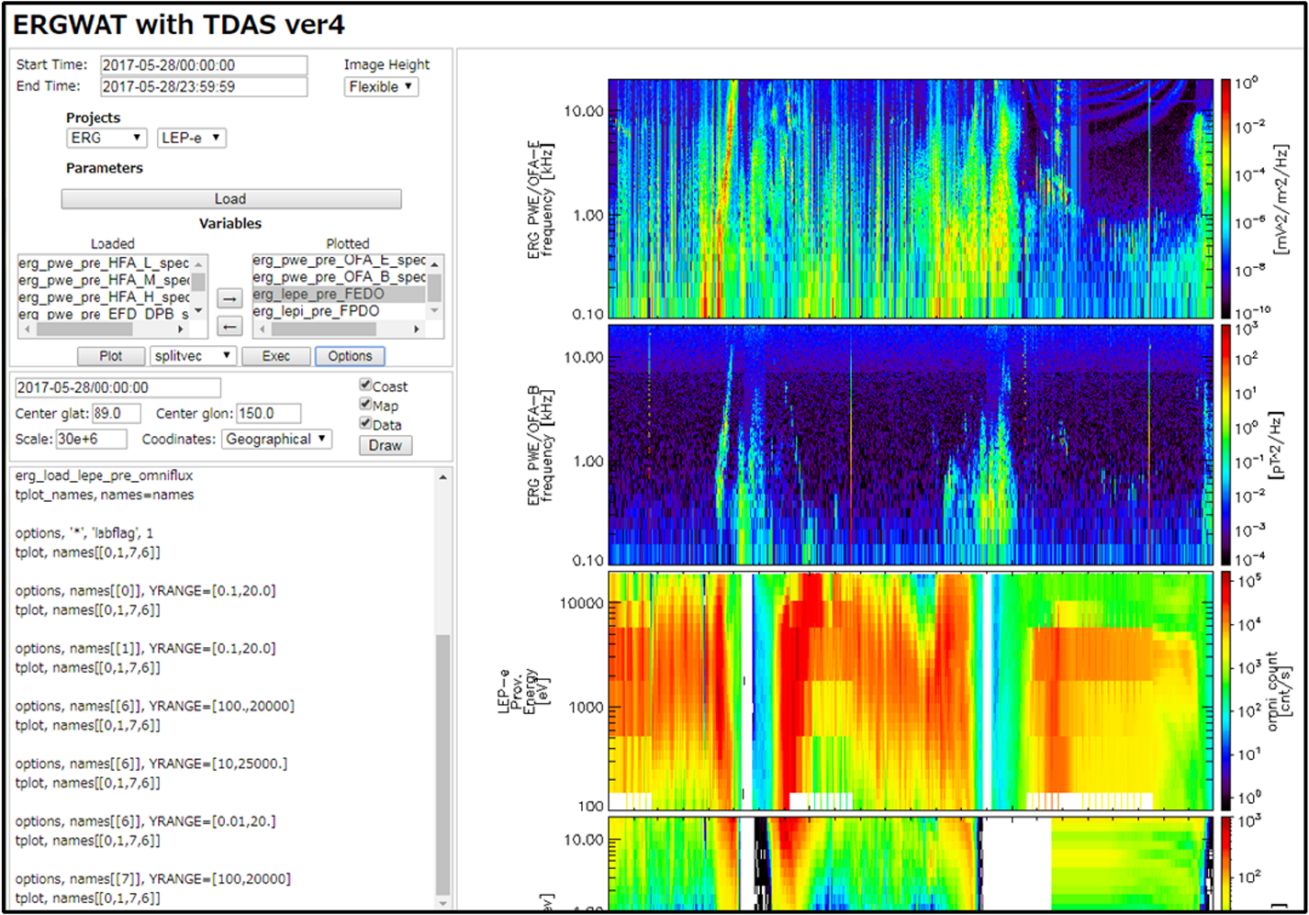
An example of SPEDAS web-services mode of use: the ERGWAT tool. This web-based form creates, in the background, SPEDAS commands that load, analyze and plot data, to the user’s specification. These commands are executed at the server institution in the SPEDAS command-line mode, and the resultant plot (in portable network graphics, PNG, form) is served on the user’s screen. The user need not know SPEDAS, or have an IDL license (it is free), and the connection up-/down-link speed requirements are modest, set by transmission of (small size) commands and PNGs, not data. The web-services mode is also useful for education and capacity building in space science, especially at remote schools or developing nations [Miyoshi et al. 2016], dramatically expanding the user base of the space physics community

The third mode of SPEDAS use is web-services. This was demonstrated and is currently in use by the ERG Web Analysis Tool, ERGWAT, of the ERG project [Umemura et al. 2017]. (Please note that a password is required to access this tool. This can be readily obtained by contacting the ERG Science Center, or the Project Scientist.) The tool is a web-based interactive data loading, visualization and analysis tool utilizing SPEDAS command-line as the engine, which is running at the server location where the licenses reside. Figure 5 is a screen-shot from the ERGWAT application, showing the user menu options on the top left, the resultant SPEDAS command-line operations and history invoked by the engine on the bottom left, and the plots on the right. The menu is simply a web-based form where the users can do the following: [1] Input necessary parameters such as date, instrument, etc., and then load them by clicking on the “Load” button, which ingests and stores the necessary files from the remote data server to the ERGWAT server, and the associated tplot variables are listed in the browser; [2] Choose variables to plot (clicking the “Plot” button) and inspect at any time in the session (and rescale size, color, etc. through the “Options” button); and [3] Analyze using SPEDAS commands such as “tdpwrspc” or “wav_data” (creating a dynamic power spectrum or a wavelet spectrum respectively) or others, through a special analysis button with a pull-down menu. The resultant spectra or other analysis products can be visualized accordingly. The users can then iterate on any of the above steps. The SPEDAS command-line operations and history serve both as confirmation of progress to the user and in order for the user to share their session details with other users or in publications. All steps are taken through the web browser, so that knowledge of IDL or SPEDAS is not required. Specifically, ERGWAT users need not be familiar with IDL to understand the state of their program’s execution or infer, from someone else’s command-line history, the steps they would need to take on the ERGWAT menu to recreate that plot. After obtaining the desired plot on the screen, the associated postscript file can be downloaded from ERGWAT via the web-browser for use in publications. The web-services mode can operate free-of-license for the user as all operations are executed at a number of licenses at the server institution.

In addition to its potential for promoting research by engaging researchers to readily conduct data analysis and obtain plots with little upfront time investment, the web-services mode has high potential for education and capacity building in space physics. This is because it requires no other infrastructure other than a web interface to conduct high-level space physics data analysis, so it is ideal for developing countries where access to high-power computers or high-speed internet may be limited. The ERG and IUGONET teams have demonstrated ERGWAT’s successful use in training sessions for space physics data analysis in India, Indonesia, Africa and South America enabling access to space physics data by hundreds of scientists around the world (Miyoshi et al. 2016). This has the potential of making space science accessible to much wider science communities world-wide.

### User Resources

Three categories of resources are available to support SPEDAS users, both at a generic (SPEDAS) level and at a project (plug-in) level: documentation, tutorials and help options.

Documentation on the SPEDAS requirements and features (including the recommended IDL license version to use and any known/resolved issues with past licenses and operating systems), newly supported functions and recent updates is available (in addition to installation instructions) under “Downloads and Installation” at http://spedas.org. Although SPEDAS routines are modified, tested and updated daily (and are released around midnight each day as a “bleeding edge” release), periodic “officially released” versions occur once or twice per year (as of the submission date of this publication the latest SPEDAS release version is 3.1). These typically follow a month-long “stress-test” of the entire library of routines (a quality-assurance, QA, program) which ensures that recent updates did not unintentionally break legacy code or cause interference with plug-ins. These official releases represent a solid foundation from which to launch future daily releases and a safer place for the new user to start from. The latest code version (updated daily) is also available from the above site. A newsletter dubbed “Tips of the month” is released to inform users at-large of the latest, most notable software patches and enhancements.

The User’s Guide (under “Documentation”) is the most general, top level documentation for SPEDAS, applicable to both command-line and GUI modes. It describes the two main modes of use with the help of simple examples. Other useful items under Documentation are useful “Crib Sheets”, the tplot concept (under “Data Model”), “Time Handling”, and particle distribution data structures (under “3D Data Structures” and “3D Data Conversion Factors”).

The most detailed documentation on command-line routines in SPEDAS and its plug-ins exists in.html format. It is available on-line for the latest (“bleeding edge”) version under “HTML documentation” in the SPEDAS wiki page. It is also available for any software installation (part of the zipped file that contains the bundled software library) as.html links accompanying those subdirectories which contain useful code. These.html files are clickable, indexed and searchable links to each individual routine’s documentation (the top-portion of the source code, containing documentation which was provided directly by the developers).

Tutorials exist in the SPEDAS wiki (http://spedas.org) “Introduction” section for many levels of users. The un-initiated will find the YouTube Channel video link “Starting with SPEDAS” most useful; it contains installation instructions and how to create one’s first plot. The “Introductory Examples” and “SPEDAS Screenshots” offer command-line and GUI examples of how to download and plot some data. Beyond that, tutorials that have been presented previously at meetings (e.g., in conjunction with the American Geophysical Union’s general assembly or at the annual GEM meetings) are shown under “SPEDAS Presentations” and represent the next step of sophistication in tutorials. They expose the user to the full complement of software capabilities. Tutorials for individual missions also exist (for THEMIS, MMS, BARREL and others) in subdirectory “older presentations”.

Beyond the aforementioned generic SPEDAS and plug-in tutorials, there exist two-dozen detailed examples (crib-sheets) in the software distribution under subdirectory “general/examples” showing how to introduce, analyze and plot SPEDAS data in command-line mode. Many mission-specific crib-sheets also exist at the top-level of each mission under a subdirectory “examples”. For instance, “projects/mms/examples/basic” contains $\sim3$ dozen examples showing basic functionality on how to introduce MMS mission instrument data and operate on it and plotting it, whereas “projects/themis/examples/advanced” includes $\sim3$ dozen examples on how to perform advanced operations, typically on multiple THEMIS instruments or spacecraft. The user can navigate through the various subdirectories for each plug-in, and use a combination of pertinent crib-sheets and.html documentation to become quickly familiar with the code, the data and their use.

Community support on SPEDAS is streamlined for tracking and resolution through email help-requests to SPEDAS_Science_Support@ssl.berkeley.edu. Those are re-directed by the software team lead to the cognizant software engineer or developer. Direct communications between user and software engineer are then encouraged. Under the GUI main menu, a Help button streamlines GUI help requests through the same channels. The user may also contact directly the developer who has been identified at the header information of the routine in question as the major (or latest) contributor to it (with cc to the above SPEDAS_Science_Support email). Such open communications are conducive to efficient problem solving, accurate information exchange and SPEDAS package improvement for the benefit of the entire community.

## SPEDAS for Developers

SPEDAS was designed as a generic software development environment for ingestion of data from distributed datasets, data analysis and plotting by general purpose space physics tools, facilitating collaborative multi-instrument, multi-mission studies. A few basic guidelines can help facilitate project participation in this environment (space mission or ground-based observatory), as explained in this section. There exist on-line examples and documentation for developers of command-line and GUI code. Additionally, several existing plug-ins (with a variety of instrument types, number of satellites and data volumes) form a good starting point for any new project to modify and build their own. The SPEDAS core team, under NASA/HQ support, facilitates such development by providing advice and experience to project software developers, and by checking for plug-in inter-compatibility through suite testing, code conflict identification and resolution, and maintenance of a stable distribution. In this section we explain how the aforementioned design principles are implemented “under the hood”, the SPEDAS personnel and software organization and management including the QA processes. We also outline the desired features for new plug-ins in order to get the full benefit of the existing infrastructure. As these are not hard requirements but facilitate the plug-in effort, they are referred to as “desirements”; the associated benefits and alternate possibilities are also presented.

### Technical Implementation

As explained earlier, SPEDAS grew out of grass roots efforts for multi-instrument analysis on FAST and multi-mission analysis on THEMIS, because of a clear need for general-purpose, platform independent, robust software in space physics using in-situ instrumentation. Once the underlying structure was in place the actual implementation for any project is generally straightforward. The most time-consuming aspect of the project development is the agreements between scientists themselves and between scientists and software engineers regarding naming conventions, call sequences, file size, versioning, processing and attributes—in other words the interfaces between data and software. Because for any given plug-in these have now been already worked out, what is “under the hood” (IDL, with dynamically linked libraries to call minor code additions in other languages) is of lower importance. A major revamp of the code in another language is therefore not as daunting. Here we describe what, in the IDL implementation, bestows SPEDAS its properties, but it should be recognized that any other language (e.g., Python) with similar device independence, CDF library support and robustness as IDL could potentially replace the SPEDAS internal engine. This possibility is discussed in Sect. 6. The discussion below explains how the current implementation (IDL-based) addresses the SPEDAS design principles listed in Sect. 2.

#### (A) Platform Independence

The majority of SPEDAS code is IDL based, and the majority of the data ingested in SPEDAS are in ISTP-compatible CDF files. As IDL has internally implemented higher level routines for accessing, interrogating, reading and writing CDF files, using SPDF-provided software for accessing such files, the platform independence of SPEDAS is thus guaranteed by the platform independence of IDL and its CDF libraries.

SPEDAS’s reliance on IDL to develop is main routines, as well as to enable new (user-provided) routines, does not preclude non-IDL code from being integrated in its arsenal using IDL provisions for externally linking C, Fortran and Python code. This happens by compiling such external code in the user’s machine (or equivalent), then placing the dynamically loadable libraries or modules (.dll or .dlm objects) in appropriate directories within the user’s IDL files so they are accessible from the IDL higher-level code. The user can do this for their own, home-grown code. The current SPEDAS version also distributes.dlls and.dlms for legacy routines, such as the Tsyganenko mapping routines [see: http://geo.phys.spbu.ru/~tsyganenko/modeling.html and Tsyganenko 2013] and associated field line tracing. To enable use by the widest possible sector of the community, the SPEDAS team has pre-compiled such codes for the most common operating systems, and is distributing the libraries/modules along with the SPEDAS software (or they can be downloaded from the SPEDAS repository).

Similar flexibility exists in data storage. Although highly recommended, CDF files are not a hard requirement for SPEDAS plug-ins. For example, THEMIS electrostatic analyzer (ESA) files [McFadden et al. 2009], the workhorse of ESA data analysis, are non-CDF, L0 (Level-0) files which are distributed as binary (even across disparate platforms) and accessed as such by IDL’s platform independent code (big-endian versus little-endian interpretation is implemented on-the-fly according to the user’s machine, along with calibration with the latest calibration parameters). The L2 ESA files, on the other hand, are derived products (calibrated and in physical units), in CDF format, for release to SPDF in a manner satisfying NASA requirements for data dissemination. For other missions too (e.g., MMS), the majority of their data are released as CDFs at their highest resolution, as L2 products (calibrated and in physical units). They have been vetted by the teams as ready-for-release, and satisfy NASA requirements and the needs of both the teams and the community for the highest quality and resolution. However, other non-CDF, platform-independent data formats are also supported by SPEDAS. These include netCDFs (e.g., GOES and ICON datasets) and FITS files (e.g., for the Solar Magnetic Activity Research Telescope, SMART, from Hida Observatory of Kyoto University, part of the IUGONET plug-in). Such standardized data format choices not only conform to NASA’s open data policy, and provide robust associated software and human resources (e.g., SPDF support, community peer support), but also facilitate the SPEDAS environment’s platform independence.

#### (B) Project Independence

**Fig. 6:**
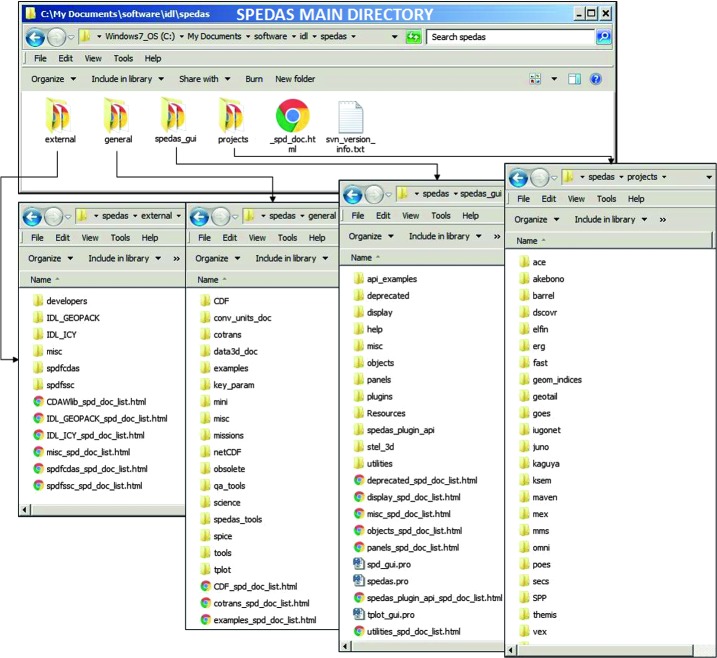
SPEDAS is comprised of library of routines in a hierarchy that reflects the code’s modularity and decentralization, the personnel management structure and the software’s development/maintenance

SPEDAS is a list of generic routines that avoid a “core” program but rely on a set of agreements to represent, load, manipulate and plot the data (as tplot or GUI variables representing physical quantities which link time, data, and associated information, as explained in Sect. 4.2). Figure 6 shows this hierarchy at the present time. Organization by directories, arranged by function (general, external, projects) and subdirectories therein, cleanly separate the general SPEDAS functions (under directory “general”) from external (to SPEDAS) but still general purpose software of significant critical mass (under “external”), and project plug-ins (under “projects”). Each plug-in is a set or routines in a separate branch of subdirectories in “projects”, which rely on the general purpose routines but are bundled together and are cleanly separated from other plug-in routines, external or generic SPEDAS contributions. GUI general routines are under “spedas_gui”; individual project GUI implementations are also under plug-in subdirectories in “projects”. This helps not only organization and searches for the users’ benefit, but simplifies code development, updates and maintenance by developers. Software maintenance with Subversion (SVN) allows seamless checkouts of temporary code renditions for testing, reverting to past versions, tracking the developer and time of an update, and managing the contributions of disparate, independent contributing teams under a common development environment. This decentralized environment enables plug-in independence. SPEDAS also promotes efficient communications across teams for software engineers to reap the benefits from past successful practices and avoid repeating past mistakes. Such communications are facilitated by weekly SPEDAS meetings between the core developers and individual mission representatives.

Generic tools exist for handling many common operations such as time conversions and coordinate transformations, including despinning. Multiple time representations (Julian day, TT2000, Epoch and Epoch16) can be converted into native time format (Unix time, or IDL time, which is UTC seconds since 1970-Jan-01 00:00:00.0) including handling of leap seconds, and is conscious of digitization errors arising when converting between them. Coordinate transformations that are platform independent reside at the general/cotrans directory and serve all projects. They convert between geophysical coordinates (Earth-, Moon- or other planet-centered) and include special tools for minimum variance and field-aligned coordinate transformations. The coordinate transformations that are platform-dependent (e.g., spacecraft-centered) reside at the plug-in directories (occasionally under a project’s own “cotrans” subdirectory), as they require position and attitude information specific to each platform. For example, “mms_cotrans” transforms between MMS sensors in body coordinates to geophysical coordinates and vice versa, and is located in that project’s subdirectories.

Extendable tools also exist for handing distribution function cuts, and unit conversions from counts to flux and phase-space density assuming the raw data are transformed in appropriate forms by the project team with the help of the core team. Robust tools for merging distributions from different instruments (spanning different energy ranges) and generic libraries for obtaining particle products, such as moments, angular spectrograms (pitch angle, gyrovelocity, azimuth or polar angle in multiple coordinate systems), energy spectrograms, and energy-angle spectra also exist. Examples of partial energy or partial angle particle products from THEMIS distribution functions are demonstrated in “thm_crib_part_products.pro”. Similar functionality for MMS and GOES exists, and other missions are working on implementing similar philosophy (e.g., ERG); those calls can be found by searching the library of available routines by typing in the command line: “*libs*, ‘*part_product*’ ”. Distribution functions are also handled with advanced tools such as the “slice2d” suite of routines in command-line mode. These functions exist for THEMIS and MMS, and can also be easily modified for other missions. A GUI interface for “slice2d” exists for THEMIS (invoked on the command line by: “*thm_ui_slice2d*” and on the generic SPEDAS GUI interface under “Tools → THEMIS particle distribution function slices”) which can form the basis for similar interfaces for other missions, though it has not yet been implemented for MMS. A generic, extendable tool, ISEE3D, has also been developed for Geotail, ERG, THEMIS and MMS [Keika et al. 2017] and has the potential of becoming a standard tool for viewing distribution functions for all missions in the future. Its only requirement is that the raw distribution function data are cast in a standardized SPEDAS IDL structure like it is on Geotail, ERG, THEMIS, and MMS.

#### (C) Command-Line, GUI, and Freeware Availability

To maximize the science return from missions it was important to offer a range of modes for data access, without increasing implementation risk and maintenance costs. The command-line mode and its associated crib-sheets offer fast and comprehensive access to the data, whereas the GUI allows quick and intuitive access to the code by the uninitiated. To avoid duplication and errors the SPEDAS software implements the GUI as an added layer on top of command-line code, relying on the same underlying routines. As explained in Sect. 4.2, tplot-variable content is accessible by GUI variable and vice-versa with a simple mapping upon user request. This allows the user to invoke operations on the data from either mode, even though operations are actually done by the same routines regardless of the mode from which they were invoked. This is possible due to the use of IDL pointers in memory, which allows IDL structures (such as tplot variables and GUI variables) to point to the data without actually accessing memory.

Two scientifically powerful freeware options are also exercised in the current SPEDAS implementation. The GUI mode is fully accessible by IDL’s Virtual Machine, which allows the user the ability to execute loading, analysis and plotting functions, as well as operate on the data using “calc”. This serves new users who do not have to learn command-line operations in order to produce highly advanced analysis in an intuitive way. This is intended to reduce the learning curve for such users, and once they become versed in analysis they can start investing more time in the more powerful command-line operations after obtaining a license. Another freeware option is the web services mode of SPEDAS execution. This also uses the GUI but does not necessitate SPEDAS installation. Rather, the only requirement is web-access as the user executes the codes on a remote server.

An intermediate option can be exercised by any project to aid analysis by the community. This entails a bundled license purchase of 50 or 100 seats, which can then be distributed to the project’s science team at a low incremental cost per user. On centralized projects the costs can be borne by the project lead institution. If that is an educational institution and students are involved in research throughout the team then the IDL license cost per user is approximately 10 times lower than a standalone license cost. For example, THEMIS owns and distributes $\sim100$ licenses to its team members and reassigns them every few years depending on need.

#### (D) Modular Code Structure

Effective data analysis relies on a modular set of codes. Introduction, data analysis, and visualization/plotting routines can thus be used to conduct effectively powerful analysis and document it or communicate it concisely. SPEDAS follows these rubrics with crib sheets or GUI documents (Sect. 4.2). Multiple threads woven within each crib-sheet or GUI document (e.g., one per product, or dataset) can create intermediate products along the way, which can be viewed and scrutinized, then combined into higher-level products for final analysis and plotting. Text-based crib-sheets or GUI documents retain records of past work, can be shared with colleagues and can be electronically published in journals referring to plots or tables of results to succinctly and accurately represent the processing done to the raw data. Aside from flexibility for the user, this code modularity is reflected also in the directory structure of SPEDAS (Fig. 6) which is conducive towards balancing the development effort as a set of different human resources: (i) a core development team (general), (ii) individual project (plug-in) software development teams (projects), (iii) other organizations contributing data and software packages, e.g., SPDF, APL (external) and (iv) the community at large (their contributions can be in individual project directories or in the general/science directory or in the external directory).

This code modularity and structure allows a distributed software engineer team, which is the most natural and efficient way of managing and evolving SPEDAS. In such a distributed team, legacy, general-purpose analysis and plotting software is maintained by the SPEDAS team and is continuously updated with new tools (many contributed by the community at large), under “general”. The onus of data queries, version tracking, downloading from remote-sites, calibration, and attribute population of a project’s code is placed on the instrument/project team itself. This team being the closest to the cognizant hardware engineers and developers is the most natural to take such tasks on. Large efforts of general utility, such as Tsyganenko models and CDAWlib functions, are bundled separately under “external”, allowing interfaces, updates and QA to be managed most efficiently with clear lines of communication and responsibility. Help from experienced SPEDAS software developers ensures quality, successful software creation. Each piece of code is internally structured to ensure documentation, comments, names and developer contact information, and, in many cases, test suites for regression testing and QA.

Software modularity is also important to ensure that user codes do not become overly complex, that the software development roles which mirror this modularity are well understood, and lines of management and responsibility are clear. Code debugging also benefits from this modularity both on the user and on the software developers’ side. This software development environment ensures optimal participation of the expert software developer and scientist user participation at a low overall cost to the project, as the above entities have a vested interest in project success, spend only as little time as possible on the code to guarantee success and are amenable to receiving help from the core developers towards the betterment of their project. Conversely, the core team utilizes its expertise to advise the project team developers in order to reduce code complexity, make the code robust and easy to peruse, modify, troubleshoot and maintain.

The modular code structure is facilitated by a distributed data environment which allows full flexibility for project teams to have their own data repository and distribution system, versioned files, the possibility of password-protected access (in case some files internal to the project need to be distributed only amongst project team members, such as e.g., for integration and test data, early mission data or uncalibrated quantities) and a clear and simple directory structure. This is implemented as file retrievals using https requests, which enables password-protection. Such protection can be implemented on a portion of the remote data, even when the majority of the dataset is open-access. User credentials are saved at the start of a session and apply for the duration of the IDL session.

The two main guidelines for facilitating such a distributed data system are that: (i) the remote data are organized in directories by quantity, then date (…project/instrument/mode/yyyy/mm/dd as appropriate), where version numbers are allowed within the same directory, signified by file extensions (e.g., filename.v2.3); and (ii) the data are internally time-ordered and have time overlaps removed (to avoid inconsistencies during plotting and data searching and for multiple files to be uniquely and accurately patched into longer time-ordered intervals per user request). A third desire, small file size, is not as critical, but is nonetheless important due to the practical consideration of file transfers across the occasionally low-speed network (at a coffee shop, airport or airplane). Files greater than $\sim10~\mbox{MB}$ should be avoided and files $\sim100~\mbox{MB}$ large become a significant liability, curtailing productivity. Conversely, small file sizes present no hardship in transfer or data analysis, since the code seamlessly patches the data from them into a single tplot variable containing contiguous data for the requested time interval. While uneven start/end boundaries and variable file sizes can be handled by standard SPEDAS routines with a layer of file-management tools, the simplest and most robust way to manage data files is to have file boundaries at fixed, known time intervals (e.g. at 10 min or 2 hour or 1 day). Such tips to projects by the core team can significantly simplify the load routines, reducing the effort by the project plug-in code developers so that the typically limited personnel resources will be better utilized for enhancement of science.

#### (E) Support Structure

SPEDAS is more than just code: it is a community of developers serving a large community of users (several wear both hats). Software evolves and companies may disappear, but the needs for analysis, visualization, and efficient science return from multi-instrument, multi-spacecraft missions are continuing and, in fact, growing. Because the community and its needs are long-term, and because human interactions (agreements) take typically longer than writing well-defined code, the underlying software itself is secondary compared to the buy-in and satisfaction of the community’s short and long-term needs. Therefore, a robust and flexible support structure is required to maintain the current code, prevent breaks or internal incompatibilities and protect it from future unexpected software industry evolution.

This is accomplished by a core team of developers, funded by NASA/HQ, in part directly and in part through NASA and international project contributions (SPDF, THEMIS, MMS, ERG, etc.). It also receives in-kind contributions (software, advice, test support) from users and other (interagency, small business) stake-holders (SPDF, ERG, IUGONET, NOAA, Cottage Systems, …). The core team releases SPEDAS code documentation for users, in the form of.html daily updates, writes and updates development guidelines, maintains current code (bug fixes) and enhances/expands it as requested by the community, and vets new code submitted by the community. The core team also supports the code developer and user community by: conducting code reviews; performing quality assurance (which entails code suite testing prior to major releases to ferret and fix new incompatibilities or bugs); regression and backwards compatibility testing; and preemptively assuring future compatibility (by interfacing pro-actively with the IDL company, Harris Geospatial Solutions, to ensure future IDL releases remain compatible with the SPEDAS software). The core team supports the community through a support line (as explained in Sect. 4), and conducts tutorials/webinars and maintains SPEDAS.org. It also interfaces with and supports project team developers on their plug-ins (including project test suites), conducts initial compatibility and beta testing for those plug-ins, and redirects community requests to them as appropriate after plug-in roll-out. Finally, it conducts advance testing of major releases, including managing testing by scientists (community volunteers who are assigned test suites to run, in real-time, and report bugs arising). As new projects become heavier users of the SPEDAS software the constitution of the core team may change to better support its most active members.

### SPEDAS Personnel Management

**Fig. 7:**
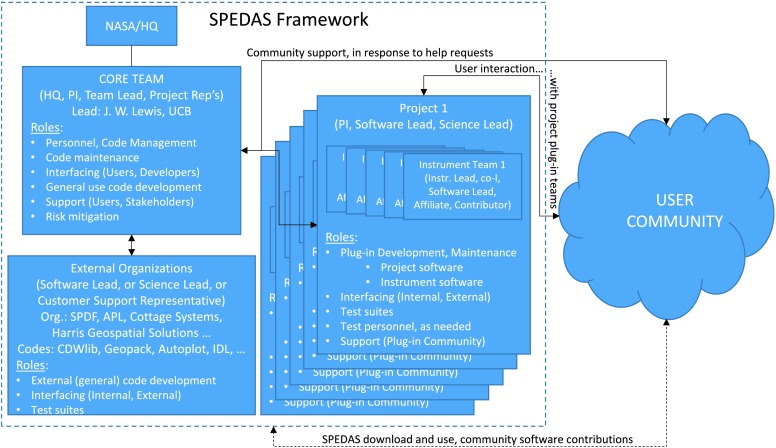
SPEDAS framework showing its management structure: teams, roles and responsibilities. Lines represent common interaction channels. The core team facilitates these interactions so they are most efficient, maintains code, supports the community at large and manages SPEDAS personnel and code, including mitigating risks from IDL version releases and software technology evolution. NASA/HQ currently holds a modest SPEDAS support contract (1.6FTE) and the remainder of the core SPEDAS personnel is contributed by individual projects

The SPEDAS framework is shown in Fig. 7. Personnel organization, roles and responsibilities, lines of communication and team compositions are also delineated therein. It is evident that personnel organization mirrors the code organization. Just like the code, which is not a central program but an inter-connected set of libraries based on a few agreements on data/time representation and file structure, the personnel too lacks a single line of authority (contractual authority delegates power to the core team for decision-making by consensus). The core team is comprised of NASA/HQ, the SPEDAS contract PI, the SPEDAS software lead and key developers funded or contributed from major projects and the main stakeholders. This team of a dozen individuals acts as a board of directors that prioritizes (based on consensus by the experts) the on-going tasks and community requests, addresses the immediate SPEDAS goals for community support and code inter-operability, and periodically assesses new features and long-term evolution as funding and time permit.

The core team meets weekly to discuss the software development status and plan future work. This includes top-level description, prioritization and assignment of new bug reports and enhancement requests; status updates on tasks in progress and of any issues or assistance needed; declaration of closures; coordination with other missions and stakeholders; and planning for upcoming software releases, conferences, tutorials and webinars. Participation of other stakeholders (e.g. science team members or software developers working on other missions) is also welcomed and encouraged, on an as-needed basis. These weekly SPEDAS management meetings mostly provide updates, assess priorities and outline possible development strategies. Prolonged, detailed technical discussions are reserved for splinters, often initiated at the weekly management meeting, in order to keep the weeklies focused and efficient.

The SPEDAS team lead and a few of the core developers, also interface on a roughly bimonthly basis with representatives from NASA’s SPDF and CDAWeb facilities. These meetings typically cover items pertaining to archival of data products for missions relevant to SPEDAS; metadata requirements and best practices; upcoming updates of CDAWeb software (CDAWlib), CDF libraries and web services that might impact SPEDAS development; and discussion of upcoming missions and data products of interest, and how SPEDAS might best support them.

As IDL versions are updated periodically by Harris Geospatial Solutions, compatibility problems can arise either within SPEDAS, or between SPEDAS and a specific operating system. In the past, the team used to discover these issues after the fact, during suite testing (in most cases), or through bug reports. The SPEDAS team would then create simple test scripts (ideally a minimal standalone script that doesn’t require any SPEDAS code) in order to reproduce the problem and pass the conditions and symptoms along to Harris Geospatial via its technical support contact. These communications would often result in suggested workarounds (SPEDAS software changes) for the affected IDL release, with the issues often being fixed in the next IDL release. Recently, however, the SPEDAS team has taken a pro-active approach and has upgraded its IDL technical support contract in order to preview impending IDL releases to perform beta-testing. Now the team executes suite testing and proactively looks for potential issues, with the goal of having them corrected before the impending IDL release is rolled-out to the public.

### SPEDAS Software Management and Quality Assurance

The SPEDAS code is under continuous development; numerous updates are made daily to the master Subversion repository. Each night a snapshot of the repository is automatically generated, zipped and released to the public as the so-called “bleeding edge”. Developers or users who think they may be affected by recent code changes can download the previous night’s release. Most users opt to download the nightly builds rather than the fully QA-ed biannual SPEDAS release, in order to take advantage of the latest features and bug fixes. Those who wish to update specific changes which took place mid-day can use their own SVN license (it is free) to perform a partial update only to the routines which just changed, the fastest and most efficient SPEDAS update mechanism. Alternatively, code updates could be mailed by the core team to affected users to replace older code in the users’ software distribution. When modifications are pervasive a new software distribution “cut” can also be started manually by the core team mid-day, if needed and requested.

Core SPEDAS developers have fairly regimented practices for clean, well-documented, and well-tested code, based on many years of experience. To avoid incompatibility between old and newly committed code, the authority to commit to the main branch of the SPEDAS distribution (the one released to the public nightly) rests with the core development team. Community contributions are submitted to the SPEDAS team, and undergo assignment to a core SPEDAS member who “vets” the code and ensures good syntax, clarity, efficiency, and conformance to the SPEDAS documentation practices. This is followed by code unit-testing in collaboration with the initiator. The simplest way for outside developers to submit code is to rely on the core SPEDAS developer assigned to their “help” case. For complex code, advanced outside developers can have more control over the code check-out process: these developers are encouraged to submit unit-test suites for future QA testing, and subject the code to as many use-cases as practical, in order to minimize the risk of “breaking the build” and inconveniencing a large number of users or fellow developers. They are also encouraged to build a separate branch off of the main SPEDAS trunk in SVN, where they can have “write-permission” and through which they can expose the code to the full SPEDAS environment for compatibility testing in tandem with the core developer assigned to help. After this code review and compatibility testing the code is submitted to the bleeding edge for further use by a community of interested users, and assuming it passes that scrutiny it is considered submitted. Further “full” QA testing with the entire distribution does not take place until the next SPEDAS release. However, this early code checkout and release process through the nightly bleeding edge has the advantage that it gets updates and bug fixes out to the community as soon as possible. This optimizes the code’s use and maximizes our ability to get feedback and identify problems early in the code’s life cycle, while ensuring that the nightly releases are stable and reliable enough to be useful in scientific research.

Versioned SPEDAS releases occur approximately biannually on a somewhat irregular schedule, as warranted by the extent of new developments and the needs of the user community. Before being officially announced, SPEDAS release software builds are put through rigorous (and time-consuming) QA process. This entails using test-suites to thoroughly exercise new and upgraded features to ensure that they operate as designed for a wide range of use cases and workflows. In the command-line mode, test-suite results are compared to known successful completion results, to ensure that changes and bug fixes did not have unexpected interactions with other parts of the system. In the GUI mode, test suites consist of a scripted series of operations for a tester to perform manually, with the expected results documented. Tests for both modes are conducted using multiple IDL versions, to ensure compatibility with older and current IDL releases. They are repeated for different operating systems, to test for platform-specific issues. Each round of tests is followed by a round of bug fixing and another cycle of testing with attention on potentially affected routines, until the code converges to a state that passes all tests. The software release is then packaged and uploaded to the SPEDAS web site. The SPEDAS wiki and documentation are updated to conform to the new release. The SPEDAS executables incorporating IDL Virtual Machine are then built for Linux, Windows, and Mac and are also updated on the web. Finally, the availability of the release is announced on the SPEDAS mailing list, GEM, SPA, and CEDAR newsletters, and the SPEDAS blog page, including links to release notes, documentation, and crib sheets illustrating new capabilities.

The QA process prior to major SPEDAS version upgrades could take up to two months, even with automated test scripts for most of the command-line mode. To cut down the duration of the QA process by a factor of two the SPEDAS team relies on scientist volunteers who run through manually executed scripts and report on the results of comparisons with known results. This is valuable community service but also benefits intermediate level users who can familiarize themselves with various SPEDAS command-line options and can build valuable professional relationships with the code development team.

### Desirements for New Plug-Ins

Creating a new SPEDAS plug-in increases the mission’s exposure to the scientific community as well as encourages correct usage of the mission data. Moreover, this is relatively straightforward. It entails: (A) creating a subdirectory of routines which load the data into tplot variables through command-line calls; and (B) building an associated GUI panel to invoke these command-line calls, and extract the tplot variables into GUI variables. Step (B) is optional but strongly recommended in order to take advantage of the free-of-charge GUI option, and to also expose the data to the largest possible sector of the community. A set of desirements make projects easy to implement in SPEDAS:

(i) IDL based code—if non-IDL then provide.dll or .dlm.
(ii) ISTP compatible CDF based data—if non-CDF then use netCDF, FITS or similar. SPEDAS can accept other (e.g. binary) data types as well, but this increases code and interface complexity.
(iii) SPEDAS-compatible directories of versioned, time-ordered files (see Sect. 5.1, item D).
(iv) Unix double precision time supported (nsec precision); currently CDFs provide msec precision through EPOCH time in seconds since AD0 (double precision) and better than nsec precision through EPOCH 16 (quadruple precision) that can also be accommodated. If higher precision than nsec is required internally, project-code needs to provide the necessary tools.
(v) File size is small enough to allow fast transfers (up to 10 MB ideally but not $> 100~\mbox{MB}$).
(vi) Distribution functions are cast into SPEDAS structures, else special project code needed.

As evident, none of these are strict requirements. However, they greatly facilitate use of existing routines by conforming to current practices. Each one can be overcome at the expense of incremental work by the plug-in development team. The most basic desirement is (i). It too can be violated by having the IDL code be an external call to Fortran, C or other code, that has been pre-compiled for a number of platforms and distributed as a.dll or .dlm. However, use of predominantly IDL code allows the user to take full advantage of the native environment of SPEDAS including the associated maintenance through future upgrades or operating system version changes.

The most important routine for a plug-in’s command-line mode is the load routine. If conforming to the standard SPEDAS practices it should specify time range, satellite and/or instrument, data type and any other identifiers specific to the program, such as operational mode, level of processing and possibly also coordinate system. Routine naming conventions also exist and, for ease of use, the plug-in’s load routine should have a name such as: “plg_load_myinstr”, where “plg” is a few (3–4) letter identifier of the project, common to all its specialized project routines, and “myinstr” is the identifier of the instrument or application identifier whose data is being loaded. This not only allows the users to quickly understand the purpose of each routine, but also avoids clashing with other SPEDAS routines or plug-in codes. This is typically accomplished by using project-specific and instrument-specific short prefixes or suffixes for all plug-in routine names. The SPEDAS wiki contains instructions (under Documentation → Developers’ Guide → Command Line) on how to use existing tools (distributed with the SPEDAS software) to achieve loading remote data. These generic tools enable SPEDAS standard load routines to identify the remote files to fulfill the load request, spawn an HTTPS (preferred), FTP, or HTTP request to download these files from a remote repository to a local data cache, and load the variables from the downloaded files into IDL as tplot variables. Given the similarity of data across space physics missions, existing load routines of a similar data type can easily be found and repurposed by the project plug-in developers with only minor changes. Such existing code can serve either as a close example for modification, or as a starting point for code development from scratch. In either case, this simplifies the development process and reduces the need for outside developers to have in-depth knowledge of the SPEDAS framework’s internal implementation details. Similarly, specialized command-line analysis and plotting routines for a new plug-in can be developed by building on existing SPEDAS tools that perform same or related functions.

GUI features for a new plug-in are optional but quite useful, as they enable loading, analysis, summary plot creation, and efficient science interactions across the community without a paid license. They also allow novice IDL users a quick access to the plug-in without much of a learning curve on the use of the plug-in’s command-line syntax. To facilitate such GUI features, which can be viewed as Application Programming Interfaces (APIs), the SPEDAS team has documented the GUI API development process at the SPEDAS wiki (under Documentation → Developers’ Guide → GUI). The standard SPEDAS software distribution provides the necessary infrastructure for such development.

Like in the command-line mode, the most important GUI feature is also data loading. The API is based on the existing “Load Data” panel. In it, the plug-in developer implements code to display and respond to the GUI controls needed to build a load request. When all the necessary parameters are filled-in, the API issues a call to the corresponding command-line load routine, then maps data from the resulting tplot variables into GUI variables. Unlike in the command mode, there is a second feature important for the GUI development of any plug-in, the configuration file. This is a simple text file, one for each plug-in, which specifies any plug-in related tabs to be added to the “Load Data” panel, “Configuration Settings” panel, the “Analysis panel”, and the “Tools” pull-down menus, along with the plug-in routine names corresponding to each tab. This configuration file is “imported” in the SPEDAS distribution simply by its placement at a specific directory. At runtime, SPEDAS searches that directory looking for, and activating all plug-in configuration files. Correspondences between GUI controls and plug-in routines are then stored internally, and the GUI controls for the new plug-in are thus created and populated. The configuration file specification is most easily done using the Configuration API (and the process is listed also in the wiki documentation). Therefore, GUI plug-in development requires a bit more detailed knowledge of SPEDAS internal implementation details than command-line-only plug-ins, but a well-documented process exists and SPEDAS core developers are also available to work closely with projects and assist with integrating their plug-ins into the SPEDAS GUI.

## SPEDAS Expansion Plans

The Heliophysics user community is best served when as many missions and data sets as possible are supported by a common software toolkit such as SPEDAS, providing a rich variety of analysis and visualization tools to the widest sector of the community. Additionally, the SPEDAS core development team strives to build interfaces with other known data ingestion or plotting packages to increase interoperability and offer alternate ways of looking at the data. The two that are presently amenable to this are Autoplot and CDAWeb. A promising new direction is also PySPEDAS, which employs a complete rewrite of the SPEDAS functions using Python. These are explained below.

First, with regards to new mission engagement, the SPEDAS team reaches out to missions as early as possible when the data project management plan is being written and the data products and analysis systems are being defined. It identifies tools or techniques that would be helpful in implementing SPEDAS support for their data analysis needs. At the very least the SPEDAS team reviews and understands the mission’s plan and makes recommendations, such that small steps can be taken which do not impede (but rather facilitate) data ingestion into SPEDAS. When implemented early such recommendations are easy to do (e.g. ISTP-compliant metadata, appropriate file sizes and directory layouts, well-defined data access policies etc.). Conversely, the SPEDAS team also strives to take any necessary steps within the SPEDAS distribution that address the needs of the new mission. For example, password-protection of a portion of the dataset was implemented for the needs of ERG and then employed by MMS for its early orbit data distribution—it is now a robust SPEDAS feature. Ideally, a token new mission is willing to go the route of an integrated analysis package for its instruments. This was the case for MMS, which benefited from an early development of its load routines and construction of crib-sheets, resulting in high science return by both the MMS team and the Heliophysics community. Towards that goal, MMS invested approximately one full-time equivalent person per year to facilitate interfacing between its instrument teams and SPEDAS, which was necessary and sufficient for a multi-spacecraft, multi-instrument mission of this size. Other projects (e.g. ERG) elected to invest into their own interfaces at the instrument software support level, and provide periodically well vetted plug-ins. For those projects, the SPEDAS core team provided support and advice through frequent communications, code reviews and beta testing. As of this writing, there are plug-ins under development to support the upcoming ICON and Parker Solar Probe missions, however the exact implementation plans are still in flux. There are also plans to develop plug-ins for additional legacy data sets: GEOTAIL, Venus Express (VEX) and Mars Express (MEX), LANL-GEO, and newly reprocessed data sets from GOES and CLUSTER.

Next, with regards to other software packages, the SPEDAS team is also proactively engaged in interfacing with them to enhance the Heliophysics user experience. Autoplot is a Java-based tool which was originally developed to query and fetch data from diverse sources identified at Heliophysics’ Virtual Observatories (VxOs) and is funded by NASA. These distributed datasets are indexed and served by metadata which adhere to the Space Physics Archive Search and Extract (SPASE) guidelines, established by the Heliophysics Data and Model Consortium (HDMC). More than a dozen Virtual Observatories pertaining to space physics data exist now, and their metadata serve as a powerful way to locate datasets serving particular quantities (e.g., magnetic fields in space) across multiple missions. Autoplot is a flexible tool for accessing and viewing these datasets. Many teams have gotten accustomed to its use for visualization of their favorite datasets. While Autoplot provides a visualization but not a data analysis environment, a combination of Autoplot and SPEDAS could be a powerful addition to the Heliophysics discipline, as it could supplement it with data analysis capability. An Autoplot-SPEDAS bridge could allow disparate Autoplot-accessed data to be analyzed together using SPEDAS and the joint analysis products to be visualized by either SPEDAS or Autoplot. Additionally, as SPEDAS current versions do not yet support queries and ingestion of VxO data, using these Autoplot capabilities as a front-end to SPEDAS could provide SPEDAS users access to more data through VxOs and new, perhaps even preferable ways of locating and importing data they are already accustomed to in their studies. Conversely, SPEDAS variables could be ported into Autoplot to provide a visualization experience they may be more familiar with. Thus, the SPEDAS team has built an easy-to-use interface to Autoplot. The interface allows any data stored in tplot variables to be sent to Autoplot using a command similar to the command that creates standard tplot windows—“tplot2ap”, and can send one or multiple data products to an Autoplot window with a single IDL command. In addition, efforts are on-going to bring data from Autoplot into SPEDAS, which will allow for interoperability between these two powerful tools. Information on how to do these operations is in the SPEDAS wiki under Documentation → Autoplot Interface.

**Fig. 8:**
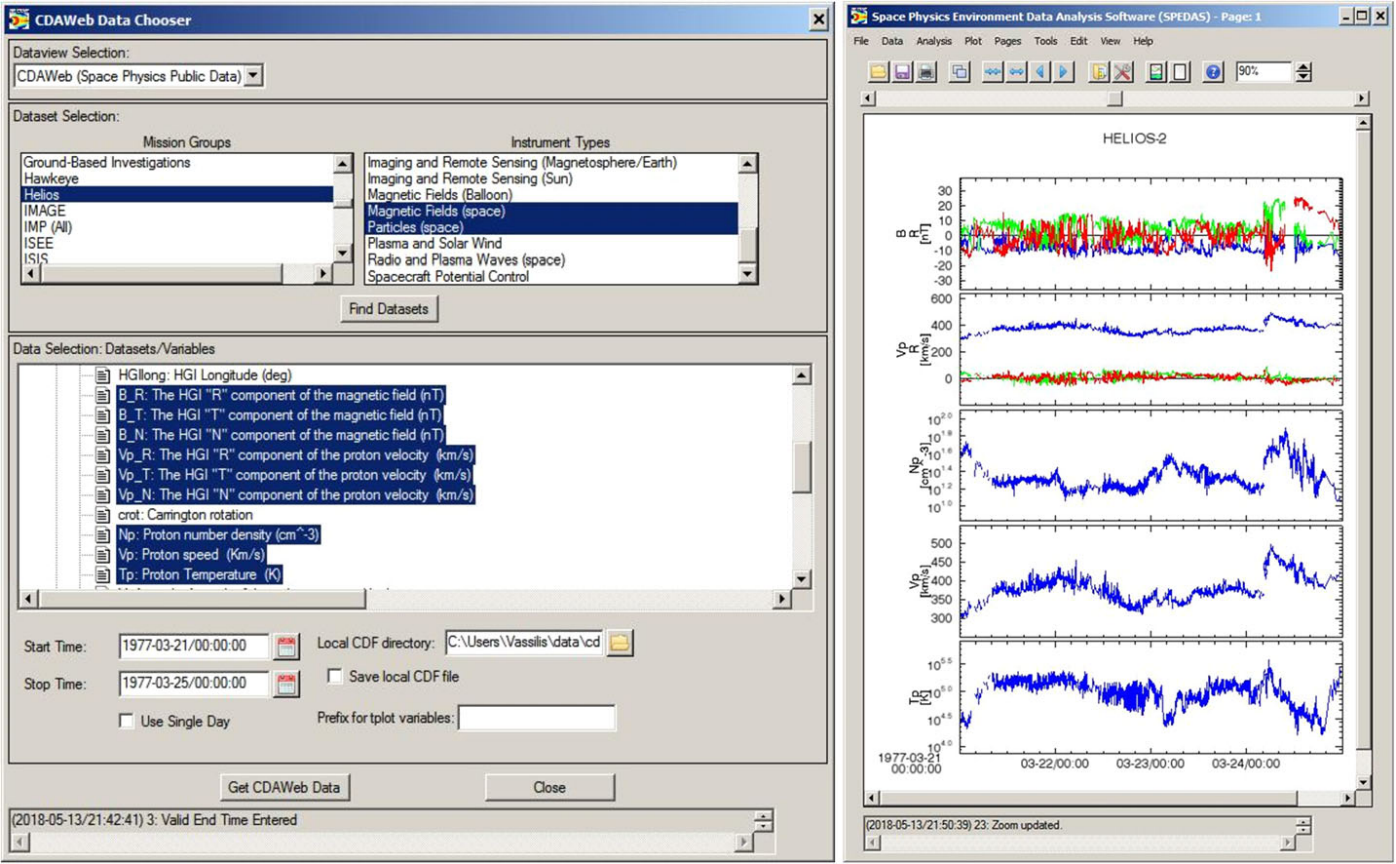
The SPEDAS interface with CDAWeb services allows the user to import data from any SPDF mission using the built-in query and data selection system of the CDAWeb front-end. This vastly expands the usability of SPEDAS in conjunction with SPDF to fetch and conduct data analysis and even more powerful visualization of the data in the user’s own machine. Left panel shows the “Load Data using CDAWeb” panel of SPEDAS. Right panel shows a plot of the quantities that were downloaded, which is accomplished with only a few mouse clicks. Further data analysis can proceed in SPEDAS GUI, or after mapping the data into tplot variables using the “Manage Tplot and GUI Variables” tab under I/O Data in the main menu

Additionally, CDAWeb, a world wide web-based plotting tool which provides access to the vast array of space missions and other products at SPDF, can also benefit from and aid SPEDAS in their common quest to better serve the Heliophysics community. Specifically, CDAWeb serves as a data query, ingestion and visualization of the SPDF data repository. This repository contains data from most US NASA missions going back to the Apollo program—the most recent ones in ISTP-compatible CDF format. The CDAWeb user-interface is hypertext markup language (HTML)-based and relies on common gateway interface (CGI) scripts to spawn IDL runs at local machines and display dynamically the data on the web. Its toolkit, a library of IDL routines to query, access, and correctly plot data stored in mission CDFs, is CDAWlib (NASA-sponsored and freely accessible). Behind the scenes is a dedicated workforce at NASA/SPDF with more than 20 years of experience. The SPEDAS team and code have greatly benefited from collaborations with this team, and has re-used elements of the CDAWlib for its purposes (e.g., time handling and CDF access). Most notably, the SPEDAS team has implemented an interface to the CDAWeb data access via the SPEDAS GUI, called: “Load Data using CDAWeb”. This brings up a session which allows the user to select mission, instrument and data product (Fig. 8, left panel) then seamlessly map the data into GUI variables and plot them with a few mouse clicks (Fig. 8, right panel). This allows a SPEDAS user to conduct science from a large array of historical missions but also from those current missions whose data may not yet be directly available as SPEDAS plug-ins.

Finally, the SPEDAS core development team is exploring the possibility of implementing some subset of SPEDAS in Python to experiment with its usefulness. Proof-of-concept tools and libraries have been already developed at CU/LASP, UCB/SSL, and UCLA, with support for loading and plotting data from the THEMIS/ARTEMIS spacecraft and several ground magnetometer networks. Under the PySPEDAS structure, “*_load_xxx” routines have been developed to access CDF files and import data to populate pytplot variables in a generic way. Pytplot variables have been demonstrated to have a similar functionality as tplot variables in SPEDAS. Currently the SPEDAS team under NASA/HQ encouragement is in the process of reaching out to other Python developers in the space physics community, in order to better coordinate the various ongoing Python software development projects and to minimize duplication of effort. There are a number of Python libraries available for reading CDF files and other data types, and then conducting standard numerical analysis and plotting. However, many SPEDAS capabilities (for example, computing moments from 3D plasma instrument data) do not exist yet in Python and will have to be developed. The advantages of Python-based code are that: (i) a new generation of scientists and programmers are already quite versed in it; (ii) it is free; (iii) it has significant general-purpose libraries associated with it; and (iv) it is insurance for NASA Heliophysics, in case IDL becomes too expensive or falters. It is expected that within a year the nucleus of a PySPEDAS system will be in place for experimentation and further contributions by a wider sector of the community, with a set of guidelines that will render such contributions most constructive.

## Other Potential Uses

In past years, distributed datasets and VxOs have enhanced data accessibility to the Heliophysics community though SPASE, whereas inter- and intra-project data distribution and interoperability through common analysis tools have been facilitated by CDAWeb, Autoplot and more recently SPEDAS. However, as datasets become larger and more widely distributed, further abstraction of the data interfaces is expected, in order to facilitate or enhance accessibility. Toward that goal, NASA’s Heliophysics community has created a framework for a common set of web services for delivering digital time series data, the Heliophysics Application Programmer’s Interface (HAPI). HAPI specifies a set of minimum but sufficient set of requirements (Data Access Specifications, currently in version 2.0.0, see: https://github.com/hapi-server/data-specification/) for a server to deliver arbitrary time series data. A few “endpoints” (access descriptors) allow clients (data users) to fully determine the data holdings and request data from the server. This specification generalizes a server’s delivery requirements to be accessed by external software engines which are cognizant of the HAPI specifications. The SPEDAS team intends to support this development with the goal of implementing mature, stable, comprehensive interfaces to the new types of servers, and especially to bridge some of the differences in metadata representation and usage among the various external tools and services.

The recent SPEDAS release supports data provision by servers which adhere to the HAPI specification through its command-line routine “hapi_load_data” as demonstrated in “crib_hapi.pro”, and its GUI button “Load_Data_using_HAPI”. HAPI usage across the Heliophysics community would further relax (obviate) the SPEDAS desirements for data providers to supply data in ISTP-compliant CDF files and in SPEDAS-friendly directory structures, as the data is queried and loaded into the user’s SPEDAS session from the data providers’ own HAPI server through the above load data procedure. In addition, HAPI support simplifies the process of creating a command-line plug-in in SPEDAS, as developers would simply need to create a wrapper-routine around the SPEDAS-provided “hapi_load_data”. This wrapper routine supplies the “hapi_load_data” subroutine the provider’s HAPI server and dataset information; the subroutine then queries the remote server, downloads the data and maps it into tplot variables. The development of a command-line routine for bringing data into SPEDAS is then simply a matter of ensuring that various plotting metadata are filled-in (e.g., default $y$-axis and $z$-axis limits on data products), documenting the data products, and ensuring the routine is well-documented and quality assured via testing. This would be ideally done by the data provider through the load routine, or can be done as a last resort even by the user, if, for example, resources do not exist at the project’s own institution to perform such documentation. The HAPI interface to arbitrary datasets has already been put to use in SPEDAS to write generic code to introduce new plug-in data to the system in a most efficient way (under Developers’ Guide → Command Line → Load Data). More information on the SPEDAS—HAPI interfaces can be found in the SPEDAS wiki (Developers’ Guide → HAPI).

Other data access specification models, similar to HAPI, also exist. By virtue of prescribing clearly defined interfaces to the data they are also easy to link with SPEDAS. Such is the example of the “DAS” model (http://das.org), currently in version 2 (DAS2, http://www-pw.physics.uiowa.edu/das2/Das2.2.2-ICD_2017-05-09.pdf) developed to support waves instruments at the University of Iowa. It is used to distribute waveform data to teams in IDL (primarily to be viewed via Autoplot) and in Python. Given the large number of projects served by this tool, the complementarity of the data being served to those that exist at SPDF and at VxO repositories, and the use of this tool for current space physics missions (e.g., NASA’s JUNO mission) the SPEDAS team is considering investing the time to create the necessary interface with those tools. However, if a HAPI interface is developed for it, this will obviate the need for the additional effort to develop DAS2.

Traditionally the planetary space physics community has been using the Planetary Data System (PDS, https://pds.nasa.gov) to serve its data, in particular its Planetary Plasma Interactions (PPI) node at IGPP/UCLA (https://pds-ppi.igpp.ucla.edu). Recently the HAPI standard has started to be implemented by the PDS team, though it is still at an experimental stage. It would then be a small effort to complete the direct inclusion of PDS data into SPEDAS and the SPEDAS team is watching these PDS developments excitedly in order to implement a SPEDAS-PDS link in the near-future.

Additionally, NASA’s Community Coordinated Modeling Center (CCMC), where users can run one of more than 25 state-of-the art simulation models of the Sun and its interaction with Earth and planets, to compare with data from their favorite events, has also started to consider serving its output using the HAPI standard. These are developed by the Space Weather Analysis System (iSWA). Presently CCMC output is in the form of plots that can hardly be overlaid on and compared directly with data. Transmission of simulation output in standardized format that can be integrated with data promises to revolutionize data-model comparisons. The SPEDAS core team stands ready to incorporate such CCMC simulation model output, in order to enable quantitative data-model comparisons and post-processing of simulation results.

## Concluding Remarks

The Heliophysics discipline has evolved considerably over the last 20 years. Multi-spacecraft, multi-instrument missions are becoming the norm. Optimal science yield from the emergent Heliophysics/Geospace System Observatory, recommended by the Decadal Survey and endorsed by NSF for enabling science far greater than the sum of its parts, demands efficient platform-independent data query, ingestion, joint analysis and visualization techniques. SPEDAS, which grew out of the community’s efforts to satisfy this growing need, has recently been placed under NASA/HQ support in recognition of its potential for optimal science analysis from past, current and future missions. Under guidance from NASA/HQ the SPEDAS team is reaching out to enable plug-ins for new missions and has created interfaces with other on-going data ingestion and visualization tools (Autoplot, CDAWeb, PySPEDAS, HAPI) in order to provide flexibility and the most familiar environment to the Heliophysics data analyst. As Heliophysics is moving towards constellation-class missions, and reliance on large arrays of ground-based datasets is also increasing (SuperDARN; Transition Region Explorer, TREX, Donovan 2017; ULTIMA, etc.) this trend for efficient analysis is going to continue. We anticipate that standardization of interfaces such as HAPI under the auspices of the HPDE will further accelerate the ease, accuracy and comprehensiveness of data distribution, and seamlessly enable the impending increase in data sources, data volume and efficiency in scientific interactions. Further, we anticipate the exchange, peer-review and publication of crib-sheets and GUI documents to become commonplace, as a means of accurately reproducing scientific information from large, disparate sets of data. In that regard, the personnel and software management structure, quality assurance, version control, implementation heritage, excellence in user support and responsiveness to community feedback that SPEDAS has introduced are expected to be far more important and enduring than the specific low level language used to implement its calls at any point in its history.

## Electronic Supplementary Material

Below are the links to the electronic supplementary material.

Figure02.pro is a command-line IDL script that when executed produces Fig. 2 in this paper. (The user must have downloaded SPEDAS 3.1 and have an IDL license.) Execution entails either compiling the code and typing the command “Figure02” in the command window, or a cut-and-paste action (line by line or multiple lines at a time) from the text body of the file (except the first line, “pro Figure02” and last line “end” which declare the crib-sheet as a subroutine) to the command window. Please note that when loading the MMS data the user will be prompted for username and password, to which they should just click “OK” for the program to continue. (Valid MMS team credentials are only necessary for accessing commissioning phase data, or lower than L2 data.) Also, please note that the present study uses ERG (Arase) data from the following file versions: for MGF: L2_v01.01; for LEPe: L2_v01_01, for LEPi: L2_v02_00, and for PWE-OFA: L2_v02_01. (PRO 5 kB)

Figure03.pro is a command-line IDL script that produces Fig. 3 in this paper. (The user must have downloaded SPEDAS 3.1, including the Tsyganenko mapping routines, Geopack, and have an IDL license. Additionally, the user must have downloaded the file midnight.sav, as explained below.) (PRO 5 kB)

midnight.sav is an “IDL save file” containing data needed to draw the midnight magnetic meridian in Fig. 3. It must be saved in the user’s directory from which Figure03.pro is run. (SAV 30 kB)

Figure04.calc.txt is a simple crib sheet that creates a new tplot variable out of a set of imported tplot variables in the GUI. It is used to create the total magnetic field from its components for the DSCOVR mission. See main text for further explanations. (TXT 1 kB)

Figure04.tgd is a GUI document file that recreates Figure 4c in this paper. To activate the user must have SPEDAS 3.1 release and open the SPEDAS GUI, then go to “File → Open SPEDAS GUI Document” and elects to “Open” this document from their system. Execution starts automatically when the document has been opened. (TGD 422 kB)

Figure04.tgt is a GUI template that changes the background color of the plot (to ciel), and inserts a title to it (“MY FIRST OVERVIEW PLOT”). The changes take effect on any subsequent plots. If the user zooms into their first plot created by the GUI document, the next plot (Page 2) will be affected by this template. Zoom in to earlier plot to experience these effects. This template was saved by the user and is intended to provide a desired “look and feel” to a plot so the user need not make these changes from scratch every time. (TGT 32 kB)

## Abbreviations

ARTEMIS: Acceleration, Reconnection, Turbulence and Electrodynamics of the Moon’s Interactions with the Sun mission (NASA)
BARREL: Balloon Array for Radiation-belt Relativistic Electron Losses (NASA)
CCMC: Community Coordinated Modeling Center
CDAWeb: SPDF’s Coordinated Data Analysis Workshop web
CDF: Common data format
DFS: Data Flow System
DSCOVR: NOAA’s Deep Space Climate Observatory mission (NOAA)
ERG: Exploration of energization and Radiation in Geospace mission (JAXA)
ERGWAT: ERG Web Analysis Tool
ESA: European Space Agency
ESA: THEMIS electrostatic analyzer
FAST: Fast Auroral Snapshot Explorer mission (NASA)
FITS: Flexible Image Transport System
GBO: Ground-based observatory
GEM: Geospace Environment Modeling
GEOTAIL: JAXA mission launched to study in Earth’s magnetotail
GOES: Geostationary Operational Environmental Satellite (NOAA)
GUI: Graphical user interface
HAPI: Heliophysics Application Programmer’s Interface
HDMC: Heliophysics Data and Model Consortium
H/GSO: Heliophysics/Geospace System Observatory
ICON: Ionospheric Connection Explorer mission (NASA)
IDL: Interactive data language
ISEE3D: Institute for Space-Earth Environmental Research (ISEE) Nagoya University’s 3D tool
ISTP: International solar terrestrial physics
iSWA: Space Weather Analysis system
IUGONET: Inter-university Upper atmosphere Global Observation NETwork
JAXA: Japan Aerospace Exploration Agency
LANL-GEO: Los Alamos National Laboratory’s Geostationary satellites (DOD)
MEX: Mars Express mission (ESA)
MMS: Magnetospheric Multiscale mission (NASA)
NCEI: National Centers for Environmental Information
NSSDC: National Space Science Data Center
NOAA: National Oceanic and Atmospheric Administration
PDS: Planetary Data System
POES: Polar Operational Environmental Satellites (NOAA)
PPI: Planetary Plasma Interactions
PSP: Parker Solar Probe
PySPEDAS: Python version of SPEDAS
QA: Quality Assurance (noun) or Quality-Assure (verb)
QSAS: Queen Mary and Westfield College, University of London’s Science Analysis System
SDDAS: Southwest Data Display and Analysis System
SDT: Science Display Tool
SMART: Solar Magnetic Activity Research Telescope
SPASE: Space Physics Archive Search and Extract
SPDF: Space Physics Data Facility
SDT: Science Display Tool
SPEDAS: Space Physics Environment Data Analysis System
SPLASH: Space Physics Line-plotting and Analysis Shell
SSL: Space Sciences Laboratory
SSW: SolarSoft (*SolarSoftWare)*
SuperDARN: Super Dual Auroral Radar Network
SVN: Subversion
ULTIMA: Ultra Large Terrestrial International Magnetic Array
TDAS: THEMIS Data Analysis System
THEMIS: Time History of Events and Macroscale Interactions during Substorms mission (NASA)
TREx: Transition Region Explorer
VEX: Venus Express mission (ESA)
VxO: Virtual observatory
